# Design, Synthesis, In Vitro and In Silico Studies of New Thiazolylhydrazine-Piperazine Derivatives as Selective MAO-A Inhibitors

**DOI:** 10.3390/molecules25184342

**Published:** 2020-09-22

**Authors:** Begüm Nurpelin Sağlık, Osman Cebeci, Ulviye Acar Çevik, Derya Osmaniye, Serkan Levent, Betül Kaya Çavuşoğlu, Sinem Ilgın, Yusuf Özkay, Zafer Asım Kaplancıklı

**Affiliations:** 1 Department of Pharmaceutical Chemistry, Faculty of Pharmacy, Anadolu University, Eskişehir 26470, Turkey; bnsaglik@anadolu.edu.tr (B.N.S.); ocebeci@gmail.com (O.C.); uacar@anadolu.edu.tr (U.A.Ç.); serkanlevent@anadolu.edu.tr (S.L.); yozkay@anadolu.edu.tr (Y.Ö.); zakaplan@anadolu.edu.tr (Z.A.K.); 2 Doping and Narcotic Compounds Analysis Laboratory, Faculty of Pharmacy, Anadolu University, Eskişehir 26470, Turkey; 3 Department of Pharmaceutical Chemistry, Faculty of Pharmacy, Zonguldak Bülent Ecevit University, Zonguldak 67600, Turkey; betul.kcavusoglu@beun.edu.tr; 4 Department of Pharmaceutical Toxicology, Faculty of Pharmacy, Anadolu University, Eskişehir 26470, Turkey; silgin@anadolu.edu.tr

**Keywords:** ADME properties, in vitro enzyme inhibition, molecular docking, monoamine oxidases, thiazolylhydrazine, piperazine

## Abstract

Monoamine oxidase (MAO) isoenzymes are very important drug targets among neurological disorders. Herein, novel series of thiazolylhydrazine-piperazine derivatives were designed, synthesized and evaluated for their MAO-A and -B inhibitory activity. The structures of the synthesized compounds were assigned using different spectroscopic techniques such as ^1^H-NMR, ^13^C-NMR and HRMS. Moreover, the prediction of ADME (Absorption, Distribution, Metabolism, Elimination) parameters for all of the compounds were performed using in silico method. According to the enzyme inhibition results, the synthesized compounds showed the selectivity against MAO-A enzyme inhibition. Compounds **3c**, **3d** and **3e** displayed significant MAO-A inhibition potencies. Among them, compound **3e** was found to be the most effective derivative with an IC_50_ value of 0.057 ± 0.002 µM. Moreover, it was seen that this compound has a more potent inhibition profile than the reference inhibitors moclobemide (IC_50_ = 6.061 ± 0.262 µM) and clorgiline (IC_50_ = 0.062 ± 0.002 µM). In addition, the enzyme kinetics were performed for compound **3e** and it was determined that this compound had a competitive and reversible inhibition type. Molecular modeling studies aided in the understanding of the interaction modes between this compound and MAO-A. It was found that compound 3e had significant and important binding property.

## 1. Introduction

Monoamine oxidases (MAOs) containing flavin adenine dinucleotide (FAD) are enzymes that catalyze the oxidative deamination of dietary amines and monoamine neurotransmitters [1]. There are two types of MAOs in mammals, MAO-A and MAO-B, defined by the cysteine amino acid (Cys406 in MAO-A and Cys397 in MAO-B) bound covalently to their co-factor FAD [2]. MAOs, which are about 70% similar at the amino acid sequence level, also have similar three-dimensional structures in which the active sites are highly conserved [3]. MAO-A and MAO-B are distinguished by the main differences, which contain details of the relevant active regions that explain their differences in substrate and inhibitor specificity [4]. Serotonin is a common MAO-A substratum, whereas 2-phenylethylamine and benzylamine are similar MAO-B substrates [5,6]. By knowing these structural differences, the rationalized drug design of isoform selective MAO inhibitors has been paved. MAO-A targeting has an antidepressant effect while limited MAO-B antagonists are used to manage Parkinson’s disease [6].

In many studies, in which our team has been involved in recent years, many thiazolylhydrazine derivatives have been shown to exhibit MAO inhibitory activity in the micromolar concentration range [7,8,9,10,11,12,13,14,15]. This study was carried out in order to further the activities of the compounds synthesized by our team and showing MAO-A activity in our previous studies. In our previous study [15], the pyrrole ring in the N1 position of the hydrazine did not contribute to the activity. Activity improved significantly with the morpholine ring replacing the pyrrole ring. In the docking studies, it was observed that the oxygen atom in the morpholine ring and the amino group in the Gly67 amino acid in the active region interact with the hydrogen bond.

**Figure 1:**
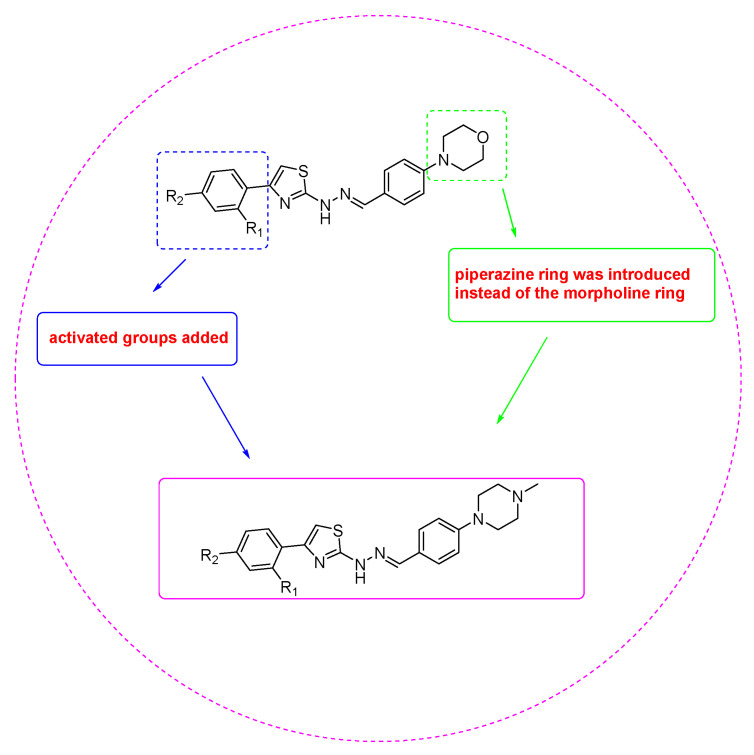
Design of target compounds.

In the light of the findings above, new thiazolylhydrazine derivatives were synthesized in this study to examine the MAO inhibitory activities. When designing the compounds to be synthesized, based on the active derivatives obtained earlier [15], the piperazine ring was introduced instead of the morpholine ring (Figure 1). Thus, the activity comparison of the piperazine ring relative to the morpholine ring can be made. The methyl group was chosen as the substituent of the piperazine ring. Depending on the contribution of the methyl group to the activity, other substituents may be tried in later studies. In addition, as the substituents of the phenyl ring in the fourth position of the thiazole ring, it was preferred activated groups (-NO_2_, -OCH_3_). Thus, the effect of these substituents on activity will be seen.

## 2. Materials and Methods

### 2.1. Chemistry

The chemicals used in the synthesis process were obtained from either Merck Chemicals (Merck KGaA, Darmstadt, Germany) or Sigma-Aldrich Chemicals (Sigma-Aldrich Corp., Louis, MO, USA). Thin layer chromatography (TLC) on silica gel 60 F254 aluminum sheets acquired from Merck (Darmstadt, Germany) has identified the reactions and purities of the compounds. The MP90 automated melting point equipment (Mettler Toledo, Ohio, USA) registered melting points of the synthesized compounds and were identified as uncorrected. ^1^H NMR and ^13^C NMR spectra were reported in DMSO-*d*_6_ with a Bruker 300 MHz and a 75 MHz digital FT-NMR spectrometer (Bruker Bioscience, Billerica, MA, USA). The patterns of splitting were defined as follows in the NMR spectra: s: singlet; d: doublet; t: triplet; m: multiplet. Coupling constants (*J*) were reported as Hertz. Mass spectra were collected using an ESI system on an LCMS-IT-TOF (Shimadzu, Kyoto, Japan).

#### 2.1.1. General Procedure for the Synthesis of the Compounds

##### Synthesis of 4-(4-Methylpiperazin-1-yl)benzaldehyde (**1**)

1-Methylpiperazine (0.04 mol, 4.00 g), 4-fluorobenzaldehyde (0.040 mol, 4.96 g) and potassium carbonate (0.048 mol, 6.62 g) were refluxed in dimethylformamide (10 mL) for 24 h. The mixture was poured into ice water and filtered. Product **1** was recrystallized from ethanol [16].

*4-(4-Methylpiperazin-1-yl)benzaldehyde* (**1**): Yield: 77%, m.p. = oily. ^1^H-NMR (300 MHz, DMSO-*d*_6_): δ = 2.21 (3H, s, -CH_3_), 2.41 (4H, t, *J* = 5.1 Hz, piperazine), 3.36 (4H, t, *J* = 5.1 Hz, piperazine), 7.03 (2H, d, *J* = 8.8 Hz, 1,4-disubstituted benzene), 7.70 (2H, d, *J* = 8.9 Hz, 1,4-disubstituted benzene), 9.71 (O=C-H). ^13^C-NMR (75 MHz, DMSO-*d*_6_): δ = 46.15, 46.77, 54.70, 113.73, 126.69, 131.92, 155.19, 190.67. HRMS (*m*/*z*): [M + H]^+^ calcd for C_12_H_16_N_2_O: 205.1335; found: 205.1328.

##### Synthesis of 2-[4-(4-Methylpiperazin-1-yl)benzylidene]hydrazinecarbothioamide (**2**)

Equal amounts of 4-(4-methylpiperazin-1-yl)benzaldehyde (**1**) (0.030 mol, 6.42 g) and thiosemicarbazide (0.030 mol, 2.87 g) were refluxed for 3 h in ethanol (80 mL). After completion of the reaction, the mixture was cooled, and the precipitated compound was filtered and recrystallized from ethanol.

*2-(4-(4-Methylpiperazin-1-yl)benzylidene)hydrazine-1-carbothioamide* (**2**): Yield: 85%, m.p. = 227–229 °C. ^1^H-NMR (300 MHz, DMSO-*d*_6_): δ = 2.20 (3H, s, -CH_3_), 2.42 (4H, t, *J* = 4.8 Hz, piperazine), 3.21 (4H, t, *J* = 4.7 Hz, piperazine), 6.92 (2H, d, *J* = 8.9 Hz, 1,4-disubstituted benzene), 7.60 (2H, d, *J* = 8.9 Hz, 1,4-Disubstituebenzene), 7.82 (1H, br s., -NH), 7.94 (1H, s, -CH=N-), 8.05 (1H, br s, -NH), 11.23 (1H, s, -NH). ^13^C-NMR (75 MHz, DMSO-*d*_6_): δ = 42.22, 47.59, 54.89, 114.87, 124.37, 128.94, 143.27, 152.42, 177.69. HRMS (*m*/*z*): [M + H]^+^ calcd for C_13_H_19_N_5_S: 278.1434; found: 278.1426.

##### Synthesis of 4-(2,4-Disubstitutedphenyl)-2-{2-[4-(4-methylpiperazin-1-yl)benzylidene]hydrazinyl} thiazoles (**3a**–**3l**)

*2-{2-[4-(4-Methylpiperazin-1-yl)benzylidene]hydrazinyl}-4-phenylthiazole* (**3a**)*:* Yield 79%, m.p. 254–255 °C. ^1^H NMR (300 MHz, DMSO-*d*_6_, ppm) δ 2.84 (3H, s, CH_3_), 3.33 (4H, br s, piperazine), 3.51 (4H, br s, piperazine), 7.05 (2H, d, *J* = 8.9 Hz, 1,4-disubstituted benzene), 7.29–7.31 (2H, m, monosubstituted benzene, thiazole), 7.40 (2H, t, *J* = 7.3 Hz, 1,4-disubstituted benzene), 7.54 (2H, d, *J* = 8.9 Hz, monosubstituted benzene), 7.85 (2H, d, *J* = 7.2 Hz, monosubstituted benzene), 7.97 (1H, s, CH=N), 12.01 (1H, s, NH). ^13^C NMR (75 MHz, DMSO-*d*_6_, ppm) δ 42.59, 45.39, 52.56, 103.74, 115.99, 125.95, 126.17, 127.90, 127.96, 129.07, 135.19, 141.87, 150.59, 150.94, 168.77. HRMS (*m/z*): [M + H]^+^ calcd for C_21_H_23_N_5_S: 378.1747; found: 378.1719.

*2-{2-[4-(4-Methylpiperazin-1-yl)benzylidene]hydrazinyl}-4-(4-methylphenyl)thiazole* (**3b**)*:* Yield 72%, m.p. 252–254 °C. ^1^H NMR (300 MHz, DMSO-*d*_6_, ppm) δ 2.30 (3H, s, CH_3_), 2.86 (3H, s, CH_3_), 3.38 (4H, br s, piperazine), 3.45 (2H, br s, piperazine), 3.88 (2H, br s, piperazine), 7.05 (2H, d, *J* = 8.9 Hz, 1,4-disubstituted benzene), 7.19 (2H, d, *J* = 8.1 Hz, 1,4-disubstituted benzene), 7.20 (1H, s, thiazole), 7.54 (2H, d, *J* = 8.9 Hz, 1,4-disubstituted benzene), 7.73 (2H, d, *J* = 8.1 Hz, 1,4-disubstituted benzene), 7.97 (1H, s, CH=N), 11.98 (1H, s, NH). ^13^C NMR (75 MHz, DMSO-*d*_6_, ppm) δ 21.28, 42.46, 45.30, 52.47, 102.82, 116.01, 125.91, 126.24, 127.89, 129.63, 132.55, 137.19, 141.79, 150.52, 150.98, 168.68. HRMS (*m/z*): [M + H]^+^ calcd for C_22_H_25_N_5_S: 392.1903; found: 392.1880.

*2-{2-[4-(4-Methylpiperazin-1-yl)benzylidene]hydrazinyl}-4-(4-methoxyphenyl)thiazole* (**3c**)*:* Yield 76%, m.p. 226–228 °C. ^1^H NMR (300 MHz, DMSO-*d*_6_, ppm) δ 2.86 (3H, s, CH_3_), 3.20 (4H, br s, piperazine), 3.53 (4H, br s, piperazine), 3.77 (3H, s, OCH_3_), 6.96 (2H, d, *J* = 8.9 Hz, 1,4-disubstituted benzene), 7.05 (2H, d, *J* = 8.9 Hz, 1,4-disubstituted benzene), 7.11 (1H, s, thiazole), 7.54 (2H, d, *J* = 8.8 Hz, 1,4-disubstituted benzene), 7.78 (2H, d, *J* = 8.8 Hz, 1,4-disubstituted benzene), 7.95 (1H, s, CH=N), 11.97 (1H, s, NH). ^13^C NMR (75 MHz, DMSO-*d*_6_, ppm) δ 42.48, 45.34, 52.50, 55.59, 101.56, 114.40, 116.02, 126.27, 127.28, 127.88, 128.07, 141.71, 150.51, 150.78, 159.18, 168.65. HRMS (*m/z*): [M + H]^+^ calcd for C_22_H_25_N_5_OS: 408.1853; found: 408.1833.

*2-{2-[4-(4-Methylpiperazin-1-yl)benzylidene]hydrazinyl}-4-(4-cyanophenyl)thiazole* (**3d**)*:* Yield 82%, m.p. 234–235 °C. ^1^H NMR (300 MHz, DMSO-*d*_6_, ppm) δ 2.86 (3H, s, CH_3_), 3.16 (4H, br s, piperazine), 3.49 (2H, br s, piperazine), 3.94 (2H, br s, piperazine), 7.06 (2H, d, *J* = 8.9 Hz, 1,4-disubstituted benzene), 7.55 (2H, d, *J* = 8.8 Hz, 1,4-disubstituted benzene), 7.62 (1H, s, thiazole), 7.86 (2H, d, *J* = 8.5 Hz, 1,4-disubstituted benzene), 7.97 (1H, s, CH=N), 8.02 (2H, d, *J* = 8.5 Hz, 1,4-disubstituted benzene), 12.09 (1H, s, NH). ^13^C NMR (75 MHz, DMSO-*d*_6_, ppm) δ 42.49, 45.31, 52.51, 107.66, 109.96, 115.99, 119.49, 126.06, 126.55, 127.99, 133.17, 139.28, 142.33, 149.27, 150.63, 169.08. HRMS (*m/z*): [M + H]^+^ calcd for C_22_H_22_N_6_S: 403.1699; found: 403.1672.

*2-{2-[4-(4-Methylpiperazin-1-yl)benzylidene]hydrazinyl}-4-(4-nitrophenyl)thiazole* (**3e**)*:* Yield 75%, m.p. 260–261 °C. ^1^H NMR (300 MHz, DMSO-*d*_6_, ppm) δ 2.87 (3H, s, CH_3_), 3.10 (4H, br s, piperazine), 3.44 (2H, br s, piperazine), 3.90 (2H, br s, piperazine), 7.05 (2H, d, *J* = 8.9 Hz, 1,4-disubstituted benzene), 7.54 (2H, d, *J* = 8.8 Hz, 1,4-disubstituted benzene), 7.68 (1H, s, thiazole), 7.98 (1H, s, CH=N), 8.09 (2H, d, *J* = 8.9 Hz, 1,4-disubstituted benzene), 8.25 (2H, d, *J* = 8.9 Hz, 1,4-disubstituted benzene), 12.12 (1H, s, NH). ^13^C NMR (75 MHz, DMSO-*d*_6_, ppm) δ 42.47, 45.27, 52.48, 108.71, 115.97, 124.56, 126.01, 126.76, 127.99, 141.17, 142.42, 146.56, 148.95, 150.64, 169.16. HRMS (*m/z*): [M + H]^+^ calcd for C_21_H_22_N_6_O_2_S: 423.1598; found: 423.1584.

*2-{2-[4-(4-Methylpiperazin-1-yl)benzylidene]hydrazinyl}-4-(4-fluorophenyl)thiazole* (**3f**)*:* Yield 69%, m.p. 247–249 °C. ^1^H NMR (300 MHz, DMSO-*d*_6_, ppm) δ 2.85 (3H, s, CH_3_), 3.33 (4H, br s, piperazine), 3.53 (4H, br s, piperazine), 7.06 (2H, d, *J* = 8.9 Hz, 1,4-disubstituted benzene), 7.20–7.26 (2H, m, 1,4-disubstituted benzene), 7.28 (1H, s, thiazole), 7.54 (2H, d, *J* = 8.8 Hz, 1,4-disubstituted benzene), 7.86–7.91 (2H, m, 1,4-disubstituted benzene), 7.96 (1H, s, CH=N), 12.01 (1H, s, NH). ^13^C NMR (75 MHz, DMSO-*d*_6_, ppm) δ 42.54, 45.36, 52.53, 103.48, 115.90 (*J* = 21.1 Hz), 115.99, 126.16, 127.92, 127.93 (*J* = 6.8 Hz), 131.82 (*J* = 2.8 Hz), 141.95, 149.91, 150.59, 162.01 (*J* = 242.7 Hz), 168.86. HRMS (*m/z*): [M + H]^+^ calcd for C_21_H_22_FN_5_S: 396.1653; found: 396.1638.

*2-{2-[4-(4-Methylpiperazin-1-yl)benzylidene]hydrazinyl}-4-(4-chlorophenyl)thiazole* (**3g**)*:* Yield 77%, m.p. 249–250 °C. ^1^H NMR (300 MHz, DMSO-*d*_6_, ppm) δ 2.85 (3H, s, CH_3_), 3.33 (4H, br s, piperazine), 3.55 (4H, br s, piperazine), 7.06 (2H, d, *J* = 8.9 Hz, 1,4-disubstituted benzene), 7.36 (1H, s, thiazole), 7.46 (2H, d, *J* = 8.6 Hz, 1,4-disubstituted benzene), 7.55 (2H, d, *J* = 8.9 Hz, 1,4-disubstituted benzene), 7.86 (2H, d, *J* = 8.6 Hz, 1,4-disubstituted benzene), 7.96 (1H, s, CH=N), 12.02 (1H, s, NH). ^13^C NMR (75 MHz, DMSO-*d*_6_, ppm) δ 42.57, 45.38, 52.57, 104.55, 115.99, 126.12, 127.66, 127.93, 129.08, 132.31, 134.05, 142.05, 149.72, 150.61, 168.90. HRMS (*m/z*): [M + H]^+^ calcd for C_21_H_22_ClN_5_S: 412.1357; found: 412.1338.

*2-{2-[4-(4-Methylpiperazin-1-yl)benzylidene]hydrazinyl}-4-(4-bromophenyl)thiazole* (**3h**)*:* Yield 85%, m.p. 253–255 °C. ^1^H NMR (300 MHz, DMSO-*d*_6_, ppm) δ 2.86 (3H, s, CH_3_), 3.19 (4H, br s, piperazine), 3.48 (2H, br s, piperazine), 3.93 (2H, br s, piperazine), 7.05 (2H, d, *J* = 8.8 Hz, 1,4-disubstituted benzene), 7.36 (1H, s, thiazole), 7.54 (2H, d, *J* = 8.9 Hz, 1,4-disubstituted benzene), 7.59 (2H, d, *J* = 8.6 Hz, 1,4-disubstituted benzene), 7.80 (2H, d, *J* = 8.6 Hz, 1,4-disubstituted benzene), 7.98 (1H, s, CH=N), 11.98 (1H, s, NH). ^13^C NMR (75 MHz, DMSO-*d*_6_, ppm) δ 42.51, 45.32, 52.53, 104.64, 116.01, 120.91, 126.19, 127.95, 127.99, 131.97, 134.42, 142.10, 149.79, 150.57, 168.92. HRMS (*m/z*): [M + H]^+^ calcd for C_21_H_22_BrN_5_S: 456.0852; found: 456.0821.

*2-{2-[4-(4-Methylpiperazin-1-yl)benzylidene]hydrazinyl}-4-([1,1’-biphenyl]-4-yl)thiazole* (**3i**)*:* Yield 83%, m.p. 275–276 °C. ^1^H NMR (300 MHz, DMSO-*d*_6_, ppm) δ 2.86 (3H, s, CH_3_), 3.34 (4H, br s, piperazine), 3.57 (4H, br s, piperazine), 7.06 (2H, d, *J* = 8.8 Hz, 1,4-disubstituted benzene), 7.34–7.39 (2H, m, monosubstituted benzene, thiazole), 7.47 (2H, t, *J* = 7.4 Hz, monosubstituted benzene), 7.56 (2H, d, *J* = 8.7 Hz, 1,4-disubstituted benzene), 7.71 (4H, d, *J* = 8.4 Hz, 1,4-disubstituted benzene), 7.94 (2H, d, *J* = 8.3 Hz, monosubstituted benzene), 7.99 (1H, s, CH=N), 12.00 (1H, s, NH). ^13^C NMR (75 MHz, DMSO-*d*_6_, ppm) δ 42.55, 45.37, 52.56, 103.98, 104.41, 116.02, 126.25, 126.55, 126.92, 127.28, 127.93, 128.49, 129.43, 134.34, 139.45, 140.12, 141.98, 150.57, 168.84. HRMS (*m/z*): [M + H]^+^ calcd for C_27_H_27_N_5_S: 454.2060; found: 454.2061.

*2-{2-[4-(4-Methylpiperazin-1-yl)benzylidene]hydrazinyl}-4-(2,4-dimethylphenyl)thiazole* (**3j**)*:* Yield 68%, m.p. 238–240 °C. ^1^H NMR (300 MHz, DMSO-*d*_6_, ppm) δ 2.28 (3H, s, CH_3_), 2.41 (3H, s, CH_3_), 2.85 (3H, s, CH_3_), 3.31 (4H, br s, piperazine), 3.52 (4H, br s, piperazine), 6.81 (1H, s, 1,2,4-trisubstituted benzene), 7.01–7.07 (4H, m, 1,4-disubstituted benzene, 1,2,4-trisubstituted benzene, thiazole), 7.48 (1H, d, *J* = 7.9 Hz, 1,2,4-trisubstituted benzene), 7.54 (2H, d, *J* = 8.7 Hz, 1,4-disubstituted benzene), 7.96 (1H, s, CH=N), 11.84 (1H, s, NH). ^13^C NMR (75 MHz, DMSO-*d*_6_, ppm) δ 21.13, 21.57, 42.57, 45,41, 52.57, 105.80, 116.05, 126.38, 126.81, 127.86, 129.58, 131.86, 132.58, 135.48, 137.04, 141.69, 150.51, 151.11, 167.78. HRMS (*m/z*): [M + H]^+^ calcd for C_23_H_27_N_5_S: 406.2060; found: 406.2021.

*2-{2-[4-(4-Methylpiperazin-1-yl)benzylidene]hydrazinyl}-4-(2,4-difluorophenyl)thiazole* (**3k**)*:* Yield 70%, m.p. 250–251 °C. ^1^H NMR (300 MHz, DMSO-*d*_6_, ppm) δ 2.79 (3H, s, CH_3_), 3.25 (4H, br s, piperazine), 3.40–3.45 (2H, m, piperazine), 3.84 (2H, br s, piperazine), 7.04 (2H, d, *J* = 8.8 Hz, 1,4-disubstituted benzene), 7.09–7.23 (2H, m, thiazole, 1,2,4-trisubstituted benzene), 7.32 (1H, td, *J_1_ =* 2.6 Hz, *J_2_* = 9.3 Hz, 1,2,4-trisubstituted benzene), 7.54 (2H, d, *J* = 8.8 Hz, 1,4-disubstituted benzene), 7.94–8.07 (2H, m, CH=N, 1,2,4-trisubstituted benzene,), 12.05 (1H, s, NH). ^13^C NMR (75 MHz, DMSO-*d*_6_, ppm) δ 42.34, 45.19, 52.26, 104.95 (t, *J* = 26.2 Hz), 107.80 (d, *J* = 14.0 Hz), 112.25 (dd, *J_1_* = 3.1 Hz, *J_2_* = 20.9 Hz), 115.96, 119.65 (dd, *J_1_* = 3.8 Hz, *J_2_* = 11.5 Hz), 126.09, 127.95, 130.83 (dd, *J_1_* = 4.8 Hz, *J_2_* = 9.3 Hz), 142.20, 143.83, 150.66, 159.98 (dd, *J_1_* = 12.1 Hz, *J_2_* = 250.2 Hz), 161.61 (dd, *J_1_* = 12.2 Hz, *J_2_* = 245.7 Hz), 168.27. HRMS (*m/z*): [M + H]^+^ calcd for C_22_H_21_Cl_2_N_5_S: 414.1558; found: 414.1527.

*2-{2-[4-(4-Methylpiperazin-1-yl)benzylidene]hydrazinyl}-4-(2,4-chlorophenyl)thiazole* (**3l**)*:* Yield 73%, m.p. >300 °C. ^1^H NMR (300 MHz, DMSO-*d*_6_, ppm) δ 2.80 (3H, s, CH_3_), 3.18–3.22 (4H, m, piperazine), 3.40–3.47 (2H, m, piperazine), 3.89–3.93 (2H, m, piperazine), 7.05 (2H, d, *J* = 8.8 Hz, 1,4-disubstituted benzene), 7.34 (1H, s, thiazole), 7.49 (1H, dd, *J_1_ =* 2.2 Hz, *J_2_* = 8.5 Hz, 1,2,4-trisubstituted benzene), 7.54 (2H, d, *J* = 8.8 Hz, 1,4-disubstituted benzene), 7.66 (1H, d, *J* = 2.1 Hz, 1,2,4-trisubstituted benzene), 7.90 (1H, d, *J* = 8.5 Hz, 1,2,4-trisubstituted benzene), 7.99 (1H, s, CH=N), 12.03 (1H, s, NH). ^13^C NMR (75 MHz, DMSO-*d*_6_, ppm) δ 42.34, 45.19, 52.26, 109.26, 115.98, 126.10, 127.95, 130.20, 131.98, 132.68, 132.69, 132.85, 142.19, 146.40, 149.92, 150.66, 168.02. HRMS (*m/z*): [M + H]^+^ calcd for C_22_H_21_Cl_2_N_5_S: 446.0967; found: 446.0936.

### 2.2. In Vitro MAO-A and MAO-B Inhibition Assay

Enzymatic testing was conducted using the current fluorometric approach declared by our research community [14,15,16,17,18]. Sigma-Aldrich (Steinheim, Germany) provided the chemicals and reagents used in the test (AmplifluTM Red (10-Acetyl-3,7-dihydroxyphenoxazine), MAO-A, MAO-B, peroxidase from horseradish, tyramine hydrochloride, H_2_O_2_, moclobemide, clorgiline and selegiline) and they were maintained by the manufacturer under the specified conditions. All pipetting processes were performed using a Biotek Precision XS robotic system (BioTek Instruments Inc., Winooski, VT, USA). Measurements were carried out by a BioTek-Synergy H1 microplate reader (BioTek Instruments Inc., Winooski, VT, USA) based on the fluorescence generated (excitation, 535 nm, emission, 587 nm) over a 30 min period, in which the fluorescence increased linearly.

In the enzymatic assay, three different daily prepared solutions were used. I) Inhibitor solutions: synthesized compounds and reference agents were prepared in 2% DMSO in 10^−3^–10^−9^ M concentrations. II) Enzyme solutions: recombinant MAO-A (0.5 U/mL) and recombinant MAO-B (0.64 U/mL) enzymes were dissolved in the phosphate buffer and final volumes were adjusted to 10 mL. III) Working solution: horseradish peroxidase (200 U/mL, 100 μL), Ampliflu™ Red (20 mM, 200 μL) and tyramine (100 mM, 200 μL) were dissolved in the phosphate buffer and the final volume was adjusted to 10 mL.

The solutions of the inhibitor (20 μL/well) and MAO-A (100 μL/well) or MAO-B (100 μL/well) were added to the flat black bottom 96-well micro test plate, and incubated at 37 °C for 30 min. After this incubation period, the reaction was started by adding a working solution (100 μL/well). The mixture was incubated at 37 °C for 30 min and the fluorescence (Ex/Em = 535/587 nm) was measured at 5 min intervals. Control experiments were carried out simultaneously by replacing the inhibitor solution with 2% DMSO (20 μL). To check the probable inhibitory effect of inhibitors on horseradish peroxidase, a parallel reading was performed by replacing enzyme solutions with 3% H_2_O_2_ solution (20 mM 100 μL/well). In addition, the possible capacity of the inhibitors to modify the fluorescence generated in the reaction mixture due to non-enzymatic inhibition was determined by mixing inhibitor and working solutions.

The specific fluorescence emission (used to obtain the final results) was calculated after subtraction of the background activity, which was determined from vials containing all components except the MAO isoforms, which were replaced by phosphate buffer (100 μL/well). Blank, control and all concentrations of inhibitors were analyzed in quadruplicate and the inhibition percentage was calculated by using the following equation:

(1) $$\%\;\mathrm{Inhibition}=\frac{\left({\mathrm{FC}}_{t2}-{\mathrm{FC}}_{t1}\right)-\left({\mathrm{FI}}_{t2}-{\mathrm{FI}}_{t1}\right)}{{\mathrm{FC}}_{t2}-{\mathrm{FC}}_{t1}}\times 100$$

where FCt_2_ is the fluorescence of a control well measured at t_2_ time, FCt_1_ is the fluorescence of a control well measured at t_1_ time, FIt_2_ is the fluorescence of an inhibitor well measured at t_2_ time and FIt_1_ is the fluorescence of an inhibitor well measured at t_1_ time.

The IC_50_ values were calculated from a dose-response curve obtained by plotting the percentage inhibition versus the log concentration with the use of *GraphPad ‘PRISM’* software (version 5.0, GraphPad Software, San Diego, CA, USA). The results were displayed as mean ± standard deviation (SD).

### 2.3. Enzyme Kinetic Studies

The same materials were used in the MAO inhibition assay. In keeping with the assay given in our previous research, the most active compound, **3e**, defined as a consequence of the MAO inhibition assay, was experienced in three independent concentrations of IC_50_/2, IC_50_ and 2(IC_50_) [14,15,16,17,18]. All processes were evaluated in quadruplicate. The results were analyzed by means of Microsoft Office Excel 2013 as Lineweaver-Burk diagrams. The Vmax values of the Lineweaver-Burk plots were replotted versus the inhibitor concentration, and the K_i_ values were determined from the x-axis intercept as K_i_. 

### 2.4. Cytotoxicity Assay

The NIH/3T3 mouse embryonic fibroblast cell line (ATCC^®^ CRL-1658 ™, London, UK) was used for cytotoxicity assays. The incubation period of NIH/3T3 cells was based on the supplier’s recommendation. NIH/3T3 cells were seeded at 1 × 10^4^ cells into each well of 96-well plates. MTT assay was carried out in accordance with the standards previously described manner [19,20]. The most effective compound **3e** was tested between 1 mM and 0.000316 mM concentrations. Inhibition % for each concentration was calculated according to the following formula and IC_50_ values were reported by plotting the % inhibition dose response curve against the compound concentrations tested [19,20,21]:

% inhibition = 100 − (mean sample × 100/mean solvent).(2)

### 2.5. Prediction of ADME Parameters

In order to predict the pharmacokinetic profiles of synthesized compounds **3a**–**3l**, QikProp 4.8 software (Schrödinger, LLC, New York, NY, USA) [22] was used, and the physicochemical parameters were calculated via the in silico method. 

### 2.6. Molecular Docking Studies

A structure-based molecular docking protocol was used to reveal the binding mechanisms of compound **3e** to the active site of the MAO-A enzyme. For this purpose, the crystal structure of MAO-A crystallized with harmine (PDB ID: 2Z5X) [23] was extracted from the Protein Data Bank database (www.pdb.org). 

The ligands’ configurations were designed using the Schrödinger Maestro [24] tool (Schrödinger, LLC, New York, NY, USA) and submitted to the Schrödinger Suite 2016 Update 2 Protein Preparation Wizard method. The ligands were processed using LigPrep 3.8 [25] to correctly detect the atom groups as well as the protonation conditions at a pH of 7.4 ± 1.0. Bond orders were assigned, and hydrogen atoms were added to the structures. The induced-fit docking (IFD) protocol [26] included in the Schrödinger Maestro interface was used to perform the IFD. 

## 3. Results and Discussion

### 3.1. Chemistry

**Scheme 1:**
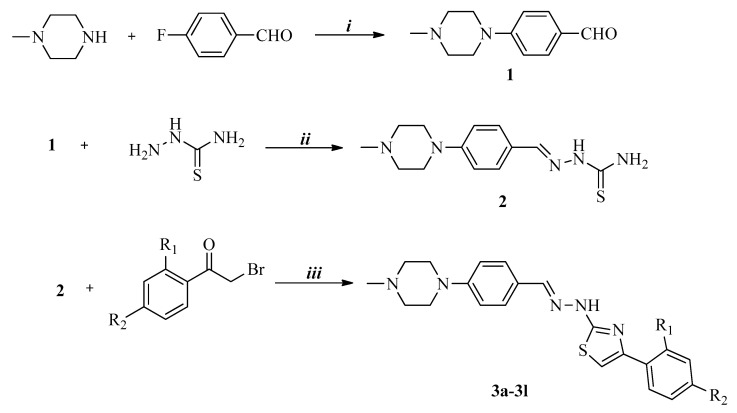
The synthetic route of the compounds (**3a**–**3l**). Reagents and conditions: (i) DMF, K_2_CO_3_, reflux, 24 h; (ii) EtOH, reflux, 3 h; (iii) EtOH, reflux, 4–8 h.

The synthesis of the compounds was completed using well established methods [14,27]. The synthetic pathways of target compounds were summarized in Scheme 1 and Table 1. Intermediate **1** was synthesized by the reaction of 1-methylpiperazine and 4-fluorobenzaldehyde in the presence of potassium carbonate. For the synthesis of compound **2**, 4-(4-methylpiperazin-1-yl)benzaldehyde (**1**) was reacted with thiosemicarbazide in ethanol. In addition, the target products **3a**–**3l** were gained by the reaction of the compound **2** and a variety of substituted phenacyl bromide derivatives. The structures and purities of the thiazole analogues were verified by ^1^H NMR, ^13^C NMR and HRMS spectral data as cited in the Supplementary Materials. The most characteristic signals observed in the ^1^H NMR spectra were those of methyl, azomethine, N-H and piperazine protons, which were present as singlets at 2.84–2.87 ppm, 7.94–7.99 ppm, 11.84–12.12 ppm and broad singlet at 3.10–3.94 ppm, respectively. In the ^13^C NMR spectra, all aliphatic and aromatic carbons were recorded at the expected regions. For all compounds, HRMS spectra corresponded with the proposed structures. 

### 3.2. MAO Inhibition Assay

All the gained thiazolylhydrazine-piperazine derivatives were evaluated for their inhibition potency against MAO isoforms using a previously described in vitro fluorometric method by our research group [14,15,16,17,18]. The enzyme activity protocol was applied in two steps according to the inhibition percentages and concentrations of the compounds. For all compounds and reference drugs, namely moclobemide, clorgiline and selegiline, the concentrations of 10^−3^ and 10^−4^ M were used in the first stage of the assay (Table 2). In this step, the reference inhibitors and compounds that showed more than 50% inhibitory activity at 10^−4^ M concentration were selected for the second step, and these compounds in question were prepared in their further concentrations by serial dilutions (ranging from 10^−5^ M to 10^−9^ M). Therefore, the half maximal inhibitory concentration (IC_50_) values of the selected compounds and reference inhibitors could be calculated, and these results are given in Table 3.

As seen in Table 2, all of the synthesized compounds demonstrated selectivity in terms of enzyme inhibitory activity on MAO-A. All of the compounds displayed a more than 50% inhibitory effect at a concentration of 10^−3^ M. This was not observed in the MAO-B enzyme inhibition results. The second stage of the enzyme activity assay was carried out through compounds **3c**, **3d** and **3e**, and their IC_50_ values were determined as seen in Table 3. It was understood that compound **3e** displayed a more potent inhibition profile than the reference inhibitors moclobemide (IC_50_ = 6.061 ± 0.262 µM) and clorgiline (IC_50_ = 0.062 ± 0.002 µM), with an IC_50_ value of 0.057 ± 0.002 µM. Moreover, compound **3e** was followed by compound **3d** as the most second active derivative with an IC_50_ value of 0.117 ± 0.004 µM. Similarly, compound **3c** showed a significant inhibition potency with an IC_50_ value of 0.188 ± 0.008 µM. 

Of the compounds effectively observed, **3d** and **3e** carried cyano and nitro groups as substituents at the phenyl ring para-position, respectively. According to the findings of the enzyme inhibition, the electron withdrawing groups such as cyano and nitro moieties were assumed to have contributed positively to enzyme inhibition ability on MAO-A enzyme.

### 3.3. Kinetic Studies of Enzyme Inhibition

**Figure 2:**
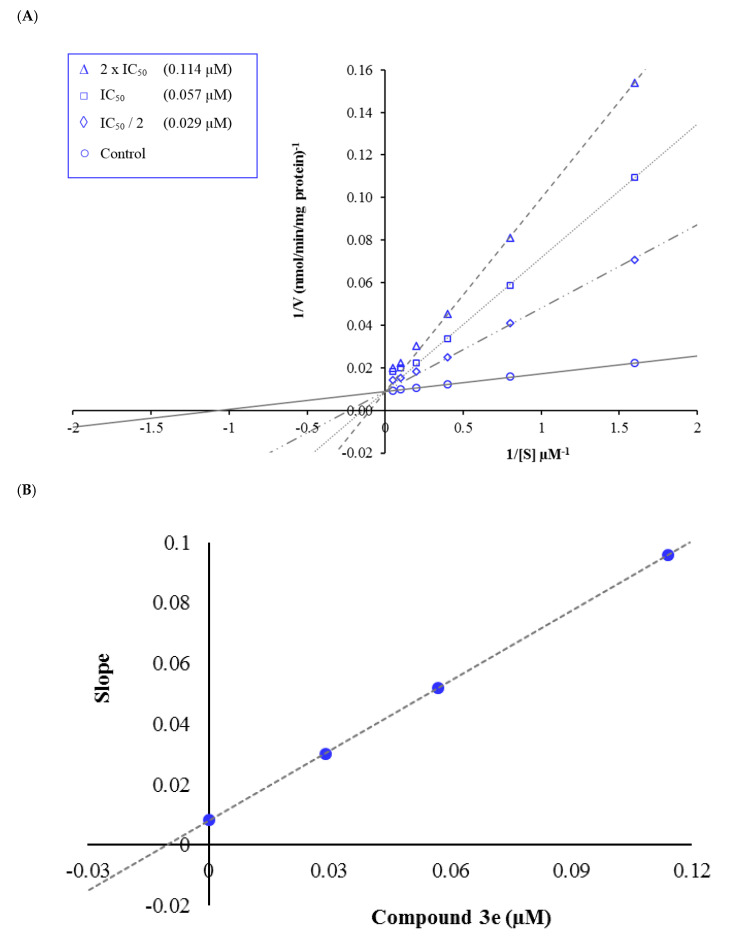
(**A**) Lineweaver-Burk plots for the inhibition of MAO-A by compound **3e**. [S], substrate concentration (µM); **V**, reaction velocity (nmol/min/mg protein). Inhibitor concentrations are shown at the left. (**B**) Secondary plot (slope, namely K_m_/V_max_ obtained from graph **A**, versus inhibitor concentrations) of compound **3e** for determining the steady-state inhibition constant (K_i_). K_i_ was calculated to be 0.011 μM.

The MAO-A inhibition mechanism was defined by conducting enzyme kinetics experiments utilizing a protocol close to that of the MAO inhibition assay. To this end, compound **3e** was included in the enzyme kinetic tests by preparation its concentrations of IC_50_/2, IC_50_ and 2(IC_50_). To estimate the type of inhibition of this compound, linear Lineweaver-Burk graphs were used. The velocity curves of the substrates were reported in the absence and presence of compound **3e**. In each case, the initial velocity measurements were collected at various concentrations of substrates (tyramine) varying from 20 μM to 0.625 μM. To measure the K_i_ (intercept on the x-axis) value of this compound, a secondary plot (Dixon plot) of the slope (K_m_/V_max_ obtained from Lineweaver-Burk graph) versus varying concentrations (0, IC_50_/2, IC_50_ and 2(IC_50_)) were generated. Figure 2 shows graphical study of the steady-state inhibition results for compound **3e**.

According to the Lineweaver-Burk plots, the type of inhibition consists of two general classes: reversible and irreversible. Mixed-type, uncompetitive, competitive and noncompetitive inhibition types are included in the reversible inhibition [14,15,16,17,18]. According to Lineweaver–Burk plots, a graph that shows parallel lines without any cross-overs is observed in the uncompetitive type of inhibition. Competitive inhibition is seen if the lines intersect on the y-axis, and the slopes and x-intercepts are different. On the contrary, non-competitive inhibition has the opposite result: the plotted lines have the same x-intercept but there are diverse slopes and y-intercepts. For mixed-type inhibition, a graph with lines that do not intersect at the x-axis or the y-axis is formed.

As seen in the Lineweaver-Burk plot of compound **3e** (Figure 2), the lines were intersected on the y-axis, and their slopes and x-intercepts were different. This observation indicated that compound **3e** was a reversible and competitive inhibitor with similar inhibition features as the substrates. The K_i_ value of compound **3e** was calculated as 0.011 μM with the help of secondary plot. 

It is known that reversible enzyme inhibition has advantages compared with the irreversible inhibition type. Non-covalent interactions, such as hydrophobic interactions, ionic bonds and hydrogen bonds between the substrate and the enzyme, are in question in the reversible inhibition and these interactions provide the forming rapidly and breaking easily of the enzyme-inhibitor complex. Reversible inhibitors often have a lower chance of adverse effects than irreversible inhibitors because of their non-covalent binding ability. Consequently, compound 3e, whose form of inhibition has been decided to be reversible and competitive, has a therapeutic value in comparison to irreversible MAO-based hydrazine inhibitors.

### 3.4. Cytotoxicity Assay

Compound **3e** displayed potent MAO-A inhibition profile and was further tested for toxicity using the MTT assay in the NIH/3T3 cell line; the IC_50_ value of this compound is shown in Table 4. Compound **3e** showed an IC_50_ value of >1000 µM against NIH/3T3 cells, which was significantly higher than its IC_50_ value (0.057 µM) against MAO-A. Consequently, compound **3e** was found to be non-cytotoxic at its effective concentration against MAO-A. This result further increases the biological importance of this compound.

### 3.5. Prediction of ADME Parameters

Inappropriate ADME (absorption, distribution, metabolism and excretion) profiles make the clinical trials of new drug development studies complex, time-consuming and costly. Thus, the assessment of pharmacokinetic profiles of new drug candidates is a vital step in the process of drug development studies [28]. Nowadays, applying in silico ADME screens can provide advantages to pick out the most promising compounds and minimize the risk of drug election in late stages [29]. Therefore, in this paper, a large amount of parameters (partition coefficient, aqueous solubility, brain/blood partition coefficient, central nervous system activity, apparent Caco-2 and MDCK cell permeability, total solvent-accessible volume, Van der Waals surface area of polar nitrogen and oxygen atoms and carbonyl carbon atoms, human oral absorption percent and drug likeness score, namely Lipinski’s rule of five and Jorgensen’s rule of three) were studied via thorough methods of virtual screening using QikProp 4.8 software [22]. The predicted parameters and their recommended values are presented in Table 5.

The drug-like quality of the compounds was tested as per the “Rule of Five” by Lipinski and the “Rule of Three” by Jorgensen [30,31,32,33]. Depending on the interaction between pharmacokinetic and physicochemical parameters, the “Rule of Five” by Lipinski and the “Rule of Three” by Jorgensen specifies the structural features found in a candidate compound, which may be a pharmaceutical product [30,31]. In Table 5, it is shown that all parameters fall inside the standard ranges. In accordance with the rules of three and five, the compounds collected (**3a**–**3l**) were in full compliance with the parameters set, since they did not cause more than one violation. Additionally, the results from compounds exhibited good CNS absorption (score of 1 and 2, namely active absorption). The investigated compounds showed medium to high cell permeability in Caco-2 and MDCK cell lines range from 98.431 to 1152.894 and 88.255 to 4373.785, respectively. These findings are very important for CNS-related drugs such as MAO-A inhibitors.

Based on the findings of the ADME parameter trials, the synthesized compounds have good and promising pharmacokinetic profiles and could be appropriate for clinical usage. 

### 3.6. Molecular Docking Studies

**Figure 3:**
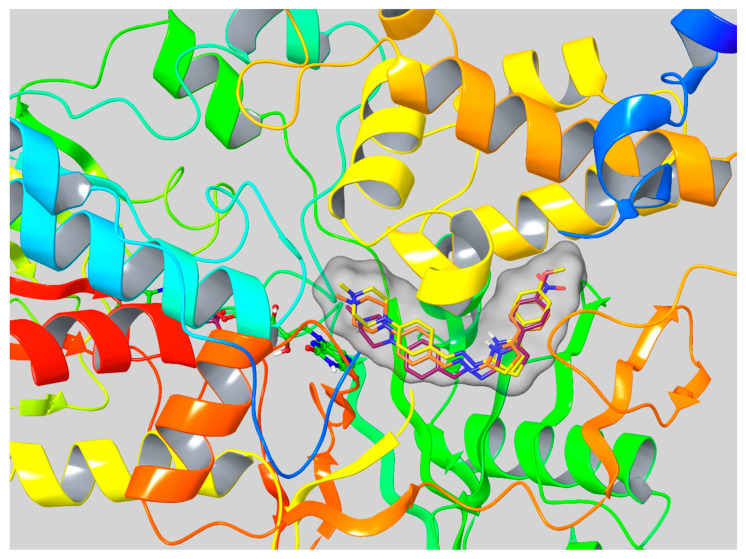
The three-dimensional (3D) poses of compounds **3c**, **3d** and **3e** in the active region of MAO-A (PDB ID: 2Z5X). The compounds are shown in a tube pattern and are yellow, maroon and orange colored, respectively.

**Figure 4:**
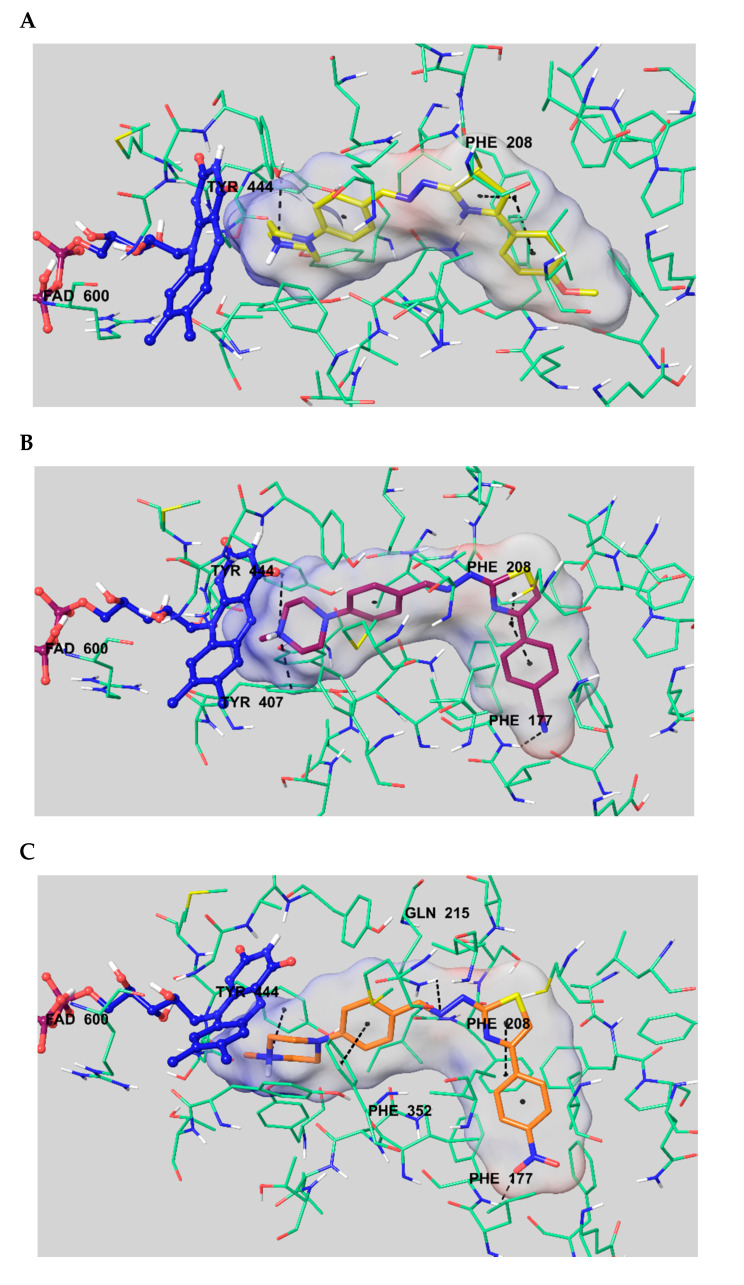
The 3D interacting modes of compound **3c**, **3d** and **3e** in the active region of MAO-A. Tube model introduce the ligands and the relevant residues in the enzyme’s active site. The flavin adenine dinucleotide (FAD) molecule is shown in a ball and stick pattern and is blue colored. (**A**) Compound 3**c** is colored = yellow. (**B**) Compound **3d** is colored maroon. (**C**) Compound **3e** is colored orange.

Compounds **3c**, **3d** and **3e** were determined to be the most effective derivatives in the series against the MAO-A enzyme as described in the MAO inhibition assay. Hence, docking studies were conducted to assess their inhibition potentials as in silico. Using the X-ray crystal structure of MAO-A (PDB ID: 2Z5X) [23], docking studies were performed, and the binding modes of these compounds were assigned. Figure 3 and Figure 4 demonstrate the docking poses of these compounds. According to Figure 3, compounds **3c**, **3d** and **3e** were correctly attached to amino acid residues filling the cavity and were positioned very close to the cofactor of FAD.

Figure 4 indicates the three-dimensional (3D) interacting modes of compounds **3c**, **3d** and **3e** in the active region of MAO-A. While examining the docking poses of these compounds, it can be obviously seen that there were many forms of interactions, such as π–π, cation-π interactions and hydrogen bond forming. Moreover, it was detected that there were the same interactions related to these compounds. A cation-π interaction occurred between the methyl substituted N atom of the piperazine ring and the phenyl of Tyr444 in all these compounds. Additionally, this N atom of compound **3d** formed another cation-π interaction with the phenyl of Tyr407. The other common interaction for all these compounds was observed between the thiazole ring and the phenyl of Phe208 by doing π–π interaction. Moreover, a similar interaction with this amino acid, Phe208, was determined by the phenyl ring near the thiazole ring in compounds **3c** and **3d**.

When analyzing the docking pose of compound **3e** (Figure 4C), it was seen that the hydrazine group in the structure was essential for polar interactions. The hydrogen bond formation was detected between the N atom of the hydrazine group and the amino of Gln215. Moreover, the phenyl ring near to the hydrazine group in compound **3e** created a π–π interaction with the phenyl of Phe352. The mentioned additional two interaction were not observed with compounds **3c** and **3d**. Therefore, these determinations were thought to be very important for the explaining of more potent enzyme inhibitory activity of compound **3e** than other compounds.

The principal structural distinction between compound **3e** and the other derivatives was the nitro group at the fourth location of the phenyl ring. The oxygen atom of the nitro group formed a hydrogen bond with the amino of Phe177. The same interaction with this amino acid was detected in the cyano group of compound **3d**. In this sense, the enzyme inhibition findings were supported by the molecular docking studies. It was thought that the substituents at this position, which served as electron withdrawing, such as the nitro and cyamo groups, strongly contributed to binding to the active site of the enzyme. This condition may also clarify why compounds **3d** and **3e** showed stronger inhibition profiles than other compounds.

## 4. Conclusions

In this paper, 12 novel thiazolylhydrazine-piperazine derivatives were reported as selective MAO-A inhibitors based on our previous studies and investigated for their inhibitory properties towards MAO enzymes using in vitro assay. Compounds **3c**, **3d** and **3e** displayed significant MAO-A inhibition profiles. The IC_50_ value of compound **3e** was lower (0.057 ± 0.002 µM) when compared to the references, moclobemide (IC_50_ = 6.061 ± 0.262 µM) and clorgiline (IC_50_ = 0.062 ± 0.002 µM). Therefore, compound **3e** was found to be the most active agent in the series. Enzyme kinetic studies revealed that the type of inhibition of this compound was identified as reversible and competitive. Moreover, the inhibitory activity on MAO-A enzyme of compound **3e** was simulated as in silico by molecular docking studies. In addition, this compound had a good pharmacokinetic profile and high BBB (blood brain barrier) penetration. Based on these findings, it was concluded that further research is needed to improve the therapeutic efficacy of this important class of compounds in the treatment of neurological disorders as MAO inhibitors. New chemical modifications can be designed based on this paper so that novel effective derivatives may be subject to future studies. Hence, studies to develop new candidates that may be effective in depression can be followed rationally.

## Figures and Tables

**Table 1:** The substituents of synthesized compounds (**3a**–**3l**).

Compound	R_1_	R_2_	Compound	R_1_	R_2_
**3a**	-H	-H	**3g**	-H	-Cl
**3b**	-H	-CH_3_	**3h**	-H	-Br
**3c**	-H	-OCH_3_	**3i**	-H	-Phenyl
**3d**	-H	-CN	**3j**	-CH_3_	-CH_3_
**3e**	-H	-NO_2_	**3k**	-F	-F
**3f**	-H	-F	**3l**	-Cl	-Cl

**Table 2:** Inhibition percentage of the synthesized compounds, moclobemide, clorgiline and selegiline against MAO-A and MAO-B enzymes.

Compounds	MAO-A % Inhibition		MAO-B % Inhibition	
	10^−3^ M	10^−4^ M	10^−3^ M	10^−4^ M
**3a**	68.137 ± 1.026	34.297 ± 0.851	32.258 ± 0.985	25.011 ± 0.721
**3b**	70.957 ± 1.114	31.456 ± 0.732	34.553 ± 0.886	27.591 ± 0.649
**3c**	92.075 ± 2.218	87.671 ± 1.874	30.336 ± 0.812	21.474 ± 0.879
**3d**	90.192 ± 2.035	84.369 ± 1.808	36.648 ± 0.903	26.044 ± 0.836
**3e**	95.314 ± 1.895	90.788 ± 1.728	39.102 ± 0.810	28.163 ± 0.791
**3f**	75.942 ± 1.235	40.666 ± 0.980	30.655 ± 0.912	24.718 ± 0.854
**3g**	73.661 ± 1.108	33.503 ± 0.833	31.250 ± 0.789	23.952 ± 0.623
**3h**	70.753 ± 1.317	37.905 ± 0.796	34.362 ± 0.824	22.104 ± 0.796
**3i**	66.197 ± 0.972	41.499 ± 0.870	30.589 ± 0.836	21.475 ± 0.809
**3j**	72.308 ± 1.033	38.122 ± 0.798	28.143 ± 0.901	20.034 ± 0.792
**3k**	79.991 ± 1.299	35.134 ± 0.833	29.573 ± 0.782	23.194 ± 0.876
**3l**	73.521 ± 1.180	40.578 ± 0.914	33.667 ± 0.991	21.373 ± 0.769
Moclobemide	94.121 ± 2.760	82.143 ± 2.691	-	-
Clorgiline	96.940 ± 1.250	91.308 ± 1.305	-	-
Selegiline	-	-	98.258 ± 1.052	96.107 ± 1.165

**Table 3:** IC_50_ values of 3c, 3d, 3e, moclobemide and clorgiline against MAO-A.

Compounds	MAO-A % Inhibition							IC_50_ (µM)
	**10^−3^ M**	**10^−4^ M**	**10^−5^ M**	**10^−6^ M**	**10^−7^ M**	**10^−8^ M**	**10^−9^ M**	
**3c**	92.075 ± 2.218	87.671 ± 1.874	75.428 ± 1.425	70.985 ± 1.095	48.336 ± 0.869	30.910 ± 0.711	21.785 ± 0.638	0.188 ± 0.008
**3d**	90.192 ± 2.035	84.369 ± 1.808	81.059 ± 1.937	78.997 ± 1.247	49.589 ± 0.811	30.637 ± 0.699	18.107 ± 0.593	0.117 ± 0.004
**3e**	95.314 ± 1.895	90.788 ± 1.728	85.025 ± 1.027	82.367 ± 1.392	63.942 ± 0.893	42.570 ± 0.835	23.018 ± 0.715	0.057 ± 0.002
Moclobemide	94.121 ± 2.760	82.143 ± 2.691	60.458 ± 2.559	36.151 ± 1.984	22.135 ± 0.337	18.166 ± 0.812	14.128 ± 0.725	6.061 ± 0.262
Clorgiline	96.940 ± 1.250	91.308 ± 1.305	87.635 ± 1.456	78.498 ± 1.024	65.235 ± 0.997	34.198 ± 0.841	22.477 ± 0.736	0.062 ± 0.002

**Table 4:** IC_50_ value of compound **3e** against the NIH/3T3 cell line.

Compound	IC_50_ (µM) NIH/3T3 Cell Line	IC_50_ (µM) *MAO-A* Enzyme
**3e**	>1000	0.057 ± 0.002

**Table 5:** Calculated ADME parameters of compounds **3a**–**3l**.

Compound	MW	RB	DM	MV	DHB	AHB	PSA	logP	logS	PCaco	logBB	PMDCK	CNS	PM	%HOA	VRF	VRT
**3a**	377.506	4	2.979	1263.957	1	7	46.680	4.092	−5.338	957.697	0.369	1032.177	1	2	100	0	0
**3b**	391.533	4	2.503	1322.897	1	7	46.680	4.393	−5.895	957.686	0.357	1032.164	1	3	100	0	1
**3c**	407.532	5	3.620	1331.741	1	7.750	55.161	4.126	−5.444	957.690	0.301	1032.168	1	3	100	0	0
**3d**	402.516	5	9.298	1330.658	1	8.500	72.475	3.323	−6.273	198.052	−0.544	187.908	1	2	87.508	0	1
**3e**	422.504	5	12.959	1346.359	1	8	95.346	3.384	−5.568	98.431	−0.912	88.255	0	3	82.432	0	0
**3f**	395.497	4	5.390	1280.070	1	7	46.684	4.326	−5.702	957.638	0.480	1866.285	2	2	100	0	1
**3g**	411.951	4	5.330	1308.076	1	7	46.682	4.583	−6.075	957.661	0.535	2546.410	2	2	100	0	1
**3h**	456.402	4	5.015	1316.988	1	7	46.682	4.660	−6.190	957.675	0.547	2737.950	2	2	100	0	1
**3i**	453.604	5	2.742	1496.172	1	7	46.680	5.687	−7.304	957.700	0.269	1032.180	1	2	100	1	1
**3j**	405.560	4	3.108	1347.245	1	7	41.791	4.557	−5.748	1152.894	0.472	1261.337	2	4	100	0	1
**3k**	413.487	4	4.457	1289.708	1	7	45.242	4.487	−5.929	994.545	0.573	2862.946	2	2	100	0	1
**3l**	446.396	4	4.564	1316.883	1	7	43.269	4.759	−5.928	1060.430	0.695	4373.785	2	2	100	0	1

**MW:** Molecular weight **RB:** Number of rotatable bonds (recommended value: 0–15) **DM:** Computed dipole moment (recommended value: 1–12.5) **MV:** Total solvent-accessible volume (recommended value: 500–2000) **DHB:** Estimated number of hydrogen bond donors (recommended value: 0–6) **AHB:** Estimated number of hydrogen bond acceptors (recommended value: 2–20) **PSA:** Van der Waals surface area of polar nitrogen and oxygen atoms and carbonyl carbon atoms (recommended value: 7–200) **logP:** Predicted octanol/water partition coefficient (recommended value: −2–6.5) **logS:** Predicted aqueous solubility (recommended value: −6.5–0.5) **PCaco:** Predicted apparent Caco-2 cell permeability (recommended value: <25 poor, >500 great) **logBB:** Predicted brain/blood partition coefficient (recommended value: −3–1.2) **PMDCK:** Predicted apparent MDCK cell permeability (recommended value: <25 poor, >500 great) **CNS:** Predicted central nervous system activity on a –2 (inactive) to +2 (active) scale (recommended value: –2 (inactive), +2 (active)) **PM:** Number of likely metabolic reactions (recommended value: 1–8) **%HOA:** Predicted human oral absorption percent (recommended value: >80% is high, <25% is poor) **VRF:** Number of violations of Lipinski’s rule of five. The rules are: MW < 500, logP < 5, DHB ≤ 5, AHB ≤ 10, Positive PSA value. **VRT:** Number of violations of Jorgensen’s rule of three. The three rules are: logS > −5.7, PCaco > 22 nm/s, PM < 7.

## Supplementary Materials

The following are available online, Figure S1. ^1^H-NMR spectra of compound **1**. Figure S2. ^13^C-NMR spectra of compound **1**. Figure S3. HRMS spectra of compound **1**. Figure S4. ^1^H-NMR spectra of compound **2**. Figure S5. ^13^C-NMR spectra of compound **2**. Figure S6. HRMS spectra of compound **2**. Figure S7. ^1^H-NMR spectra of compound **3a**. Figure S8. ^13^C-NMR spectra of compound **3a**. Figure S9. HRMS spectra of compound **3a**. Figure S10. ^1^H-NMR spectra of compound **3b**. Figure S11. ^13^C-NMR spectra of compound **3b**. Figure S12. HRMS spectra of compound **3b**. Figure S13. ^1^H-NMR spectra of compound **3c**. Figure S14. ^13^C-NMR spectra of compound **3c**. Figure S15. HRMS spectra of compound **3c**. Figure S16. ^1^H-NMR spectra of compound **3d**. Figure S17. ^13^C-NMR spectra of compound **3d**. Figure S18. HRMS spectra of compound **3d**. Figure S19. ^1^H-NMR spectra of compound **3e**. Figure S20. ^13^C-NMR spectra of compound **3e**. Figure S21. HRMS spectra of compound **3e**. Figure S22. ^1^H-NMR spectra of compound **3f**. Figure S23. ^13^C-NMR spectra of compound **3f**. Figure S24. HRMS spectra of compound **3f**. Figure S25. ^1^H-NMR spectra of compound **3g**. Figure S26. ^13^C-NMR spectra of compound **3g**. Figure S27. HRMS spectra of compound **3g**. Figure S28. ^1^H-NMR spectra of compound **3h**. Figure S29. ^13^C-NMR spectra of compound **3h**. Figure S30. HRMS spectra of compound **3h**. Figure S31. ^1^H-NMR spectra of compound **3i**. Figure S32. ^13^C-NMR spectra of compound **3i**. Figure S33. HRMS spectra of compound **3i**. Figure S34. ^1^H-NMR spectra of compound **3j**. Figure S35. ^13^C-NMR spectra of compound **3j**. Figure S36. HRMS spectra of compound **3j**. Figure S37. ^1^H-NMR spectra of compound **3k**. Figure S38. ^13^C-NMR spectra of compound **3k**. Figure S39. HRMS spectra of compound **3k**. Figure S40. ^1^H-NMR spectra of compound **3l**. Figure S41. ^13^C-NMR spectra of compound **3l**. Figure S42. HRMS spectra of compound **3l**.
