# Supplementary material for: Design, Synthesis, In Vitro and In Silico Studies of New Thiazolylhydrazine-Piperazine Derivatives as Selective MAO-A Inhibitors
Source: Molecules. 2020 Sep 22;25(18):4342. doi: 10.3390/molecules25184342 (PMC7571065; doi:10.3390/molecules25184342)
Supplement: Supplementary file 1 [file molecules-25-04342-s001.pdf]

SUPPORTING INFORMATION

Design, Synthesis, In Vitro and In Silico Studies of New Thiazolylhydrazine-Piperazine Derivatives as Selective MAO-A Inhibitors

Begüm Nurpelin Sağlık [1,2], Osman Cebeci [1], Ulviye Acar Çevik [1,2], Derya Osmaniye [1,2*], Serkan Levent [1,2], Betül Kaya Çavuşoğlu [3], Sinem Ilgın [4], Yusuf Özkay [1,2] and Zafer Asım Kaplancıklı [1]

[1] Department of Pharmaceutical Chemistry, Faculty of Pharmacy, Anadolu University, 26470 Eskişehir, Turkey
[2] Doping and Narcotic Compounds Analysis Laboratory, Faculty of Pharmacy, Anadolu University, 26470 Eskişehir, Turkey
[3] Department of Pharmaceutical Chemistry, Faculty of Pharmacy, Zonguldak Bülent Ecevit University, 67600 Zonguldak, Turkey
[4] Department of Pharmaceutical Toxicology, Faculty of Pharmacy, Anadolu University, 26470 Eskişehir, Turkey
* Correspondence: dosmaniye@anadolu.edu.tr; Tel.: +90-222-335-0580/3778

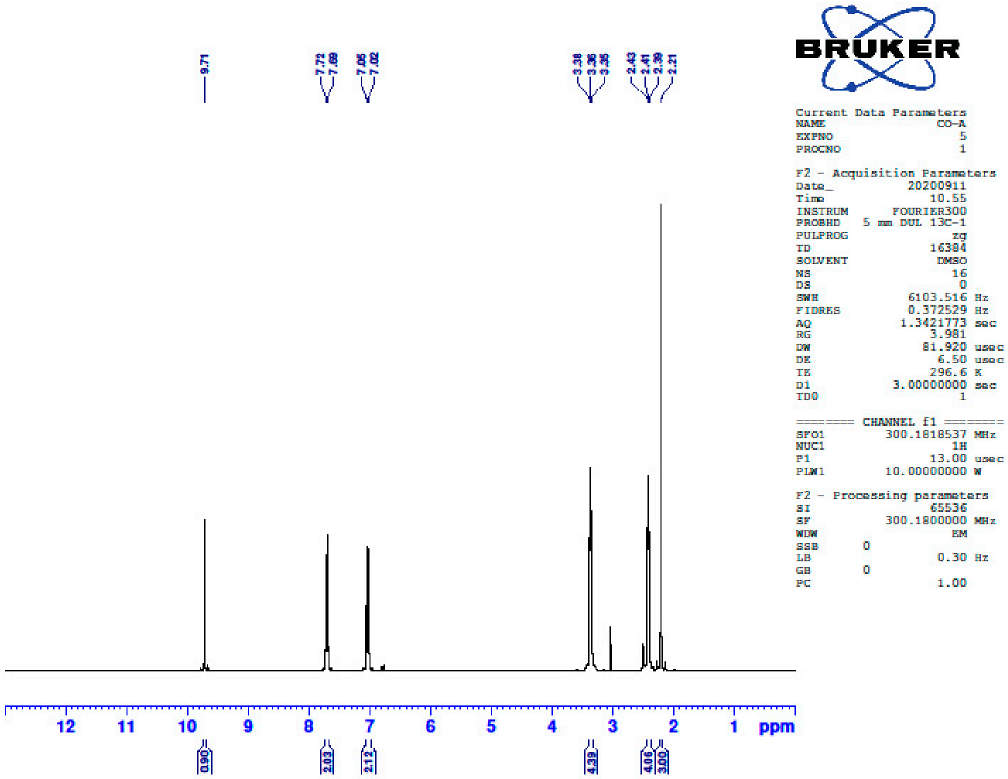

Figure S1. ^{1}H-NMR spectra of compound **1**

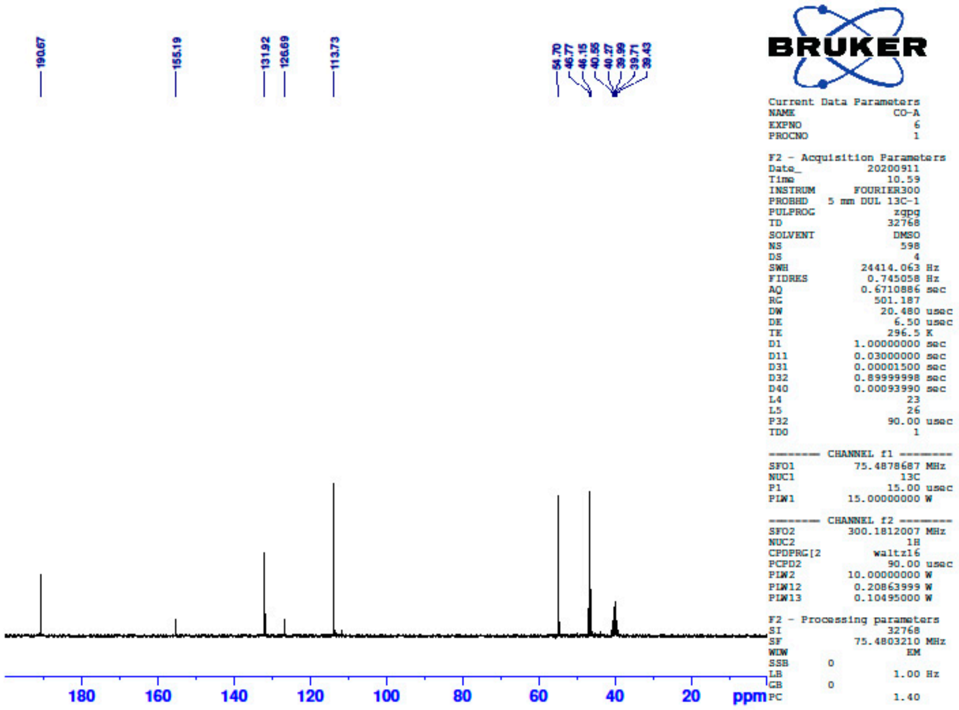

Figure S2. ^{13}C-NMR spectra of compound **1**

Data File: C:\LabSolutions\Data\Analiz\Serkan\CO-B_02.lcd

Elmt	Val.	Min	Max	Elmt	Val.	Min	Max	Elmt	Val.	Min	Max	Elmt	Val.	Min	Max	Use Adduct
H	1	5	35	O	2	0	5	S	2	0	1	Ru	2	0	0	H
C	4	9	35	F	1	0	0	Cl	1	0	0	Pd	2	0	0	
N	3	0	5	P	3	0	0	Br	1	0	0	I	3	0	0	

Error Margin (ppm): 5
HC Ratio: unlimited
Max Isotopes: 3
MSn Iso RI (%): 10.00
DBE Range: 5.0 - 20.0
Apply N Rule: yes
Isotope RI (%): 1.00
MSn Logic Mode: AND
Electron Ions: both
Use MSn Info: yes
Isotope Res: 9000
Max Results: 100

Event#: 1 MS(E+) Ret. Time : 10.133 Scan# : 1521

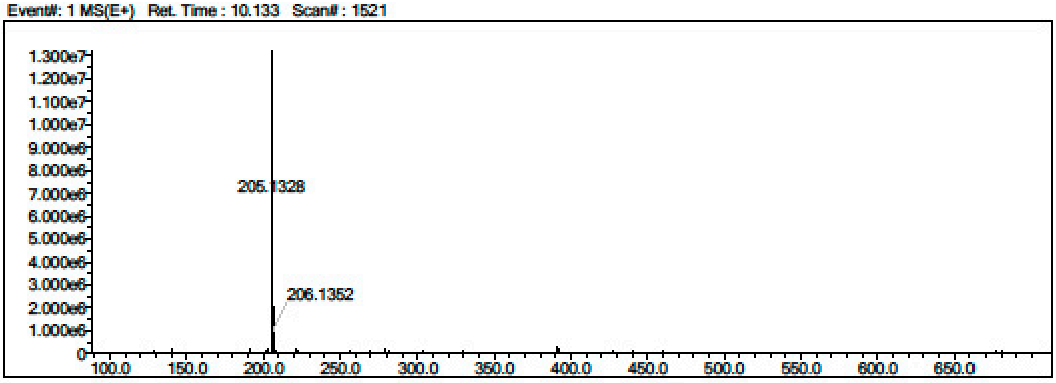

Measured region for 205.1328 m/z

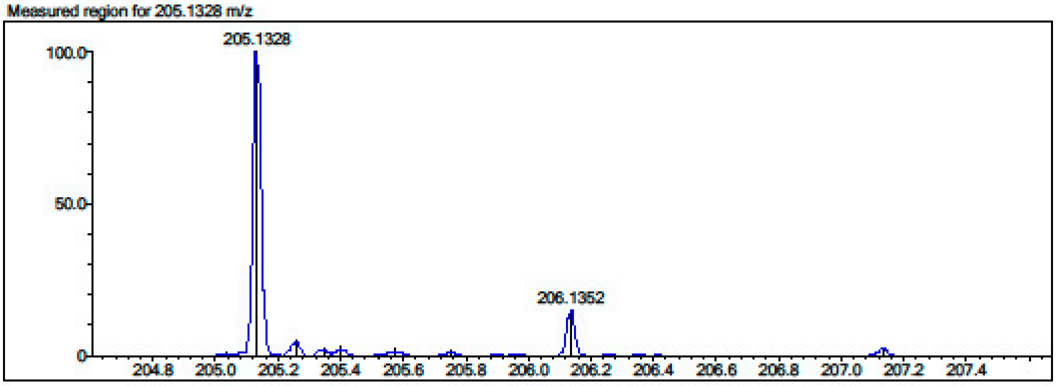

C12 H16 N2 O [M+H]+ : Predicted region for 205.1335 m/z

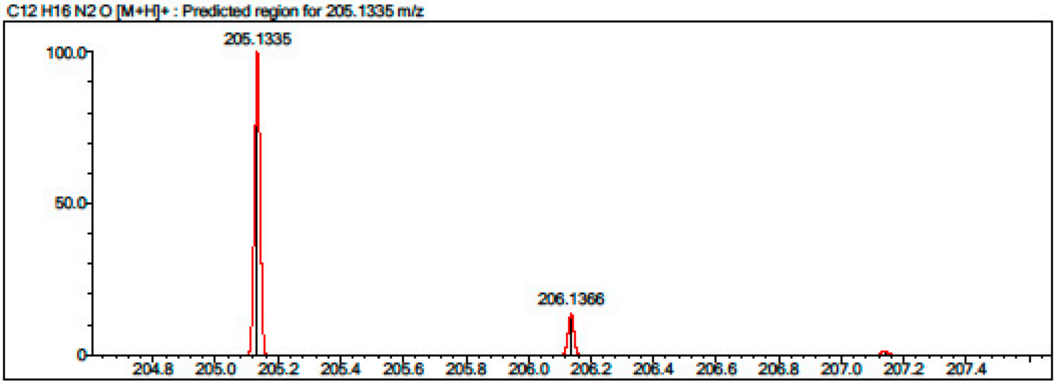

Rank	Score	Formula (M)	Ion	Meas. m/z	Pred. m/z	Df. (mDa)	Df. (ppm)	Iso	DBE
1	67.98	C12 H16 N2 O	[M+H]+	205.1328	205.1335	-0.7	-3.41	72.34	6.0

Figure S3. HRMS spectra of compound **1**

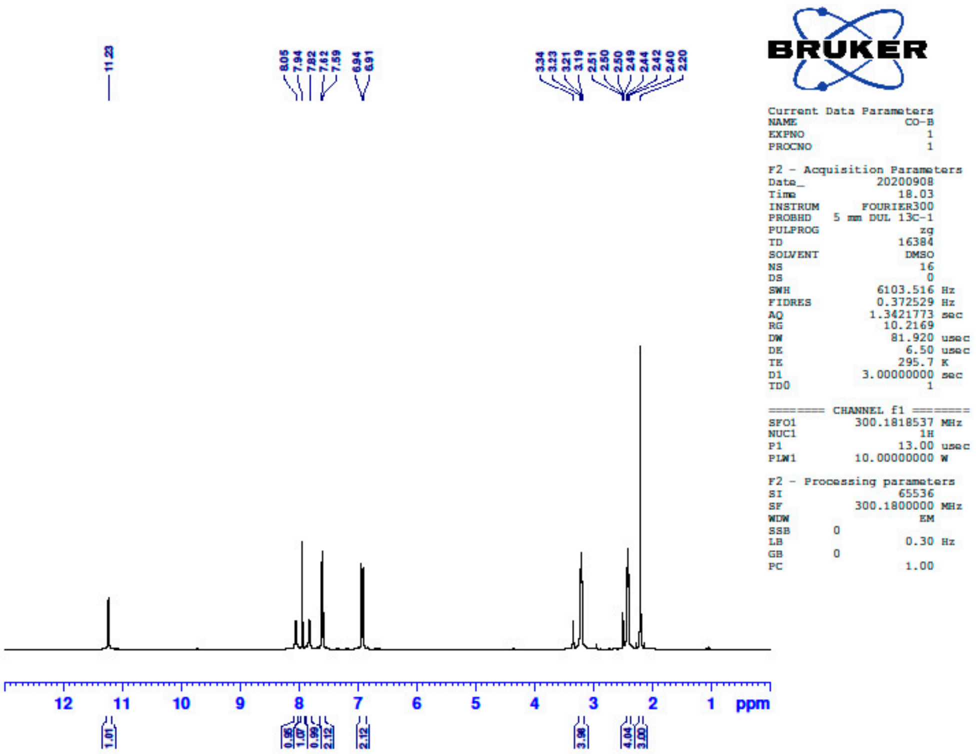

Figure S4. ^{1}H-NMR spectra of compound **2**

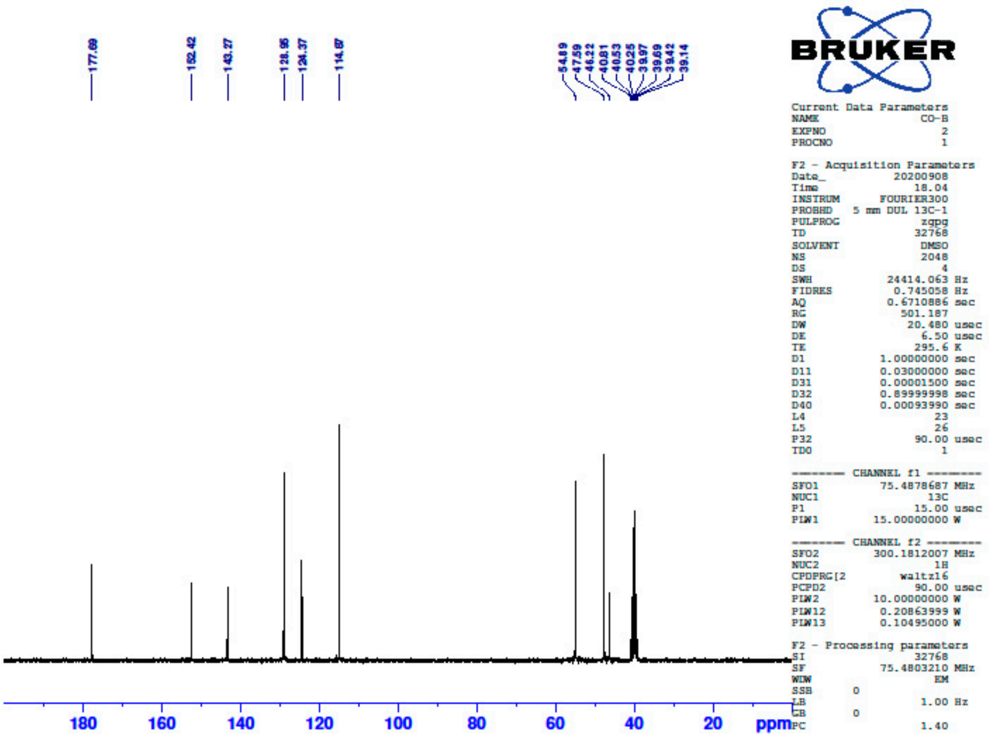

Figure S5. ^{13}C-NMR spectra of compound **2**

Data File: C:\LabSolutions\Data\Analiz\Serkan\CO-A_01.lcd

Elmt	Val.	Min	Max	Elmt	Val.	Min	Max	Elmt	Val.	Min	Max	Elmt	Val.	Min	Max	Use Adduct
H	1	5	35	O	2	0	5	S	2	0	1	Ru	2	0	0	H
C	4	9	35	F	1	0	0	Cl	1	0	0	Pd	2	0	0	
N	3	0	5	P	3	0	0	Br	1	0	0	I	3	0	0	

Error Margin (ppm): 5
HC Ratio: unlimited
Max Isotopes: 3
MSn Iso RI (%): 10.00

DBE Range: 5.0 - 20.0
Apply N Rule: yes
Isotope RI (%): 1.00
MSn Logic Mode: AND

Electron Ions: both
Use MSn Info: yes
Isotope Res: 9000
Max Results: 100

Event#: 1 MS(E+) Ret. Time : 1.560 Scan# : 235

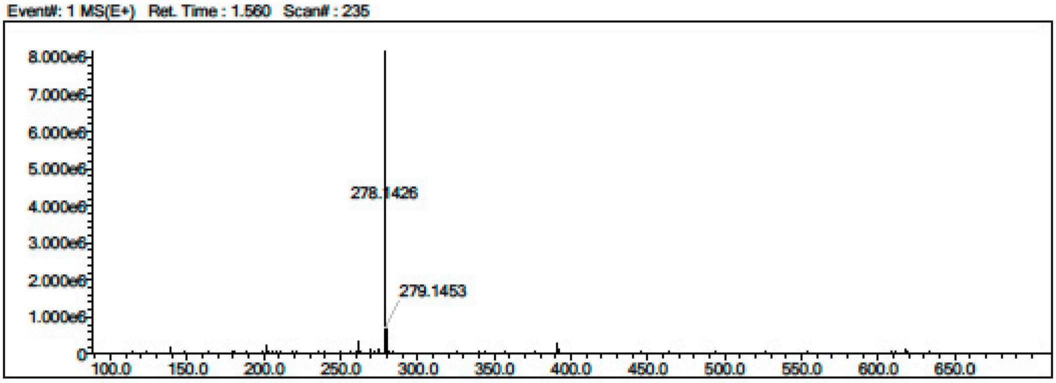

Measured region for 278.1426 m/z

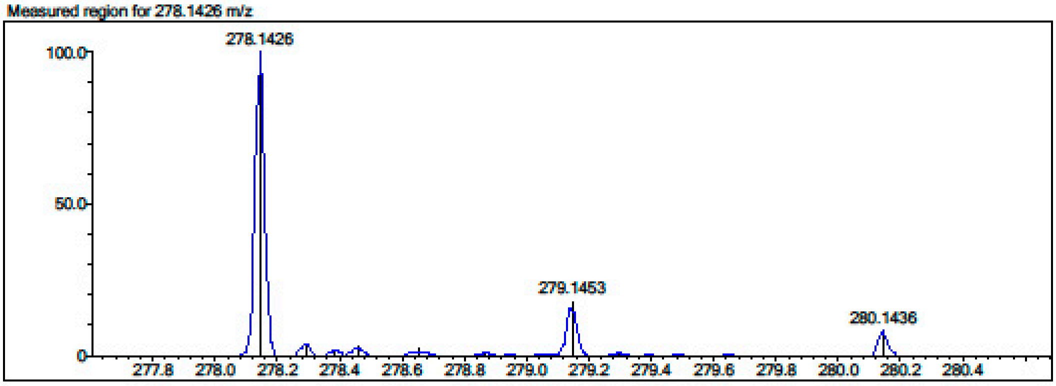

C13 H19 N5 S [M+H]+ : Predicted region for 278.1434 m/z

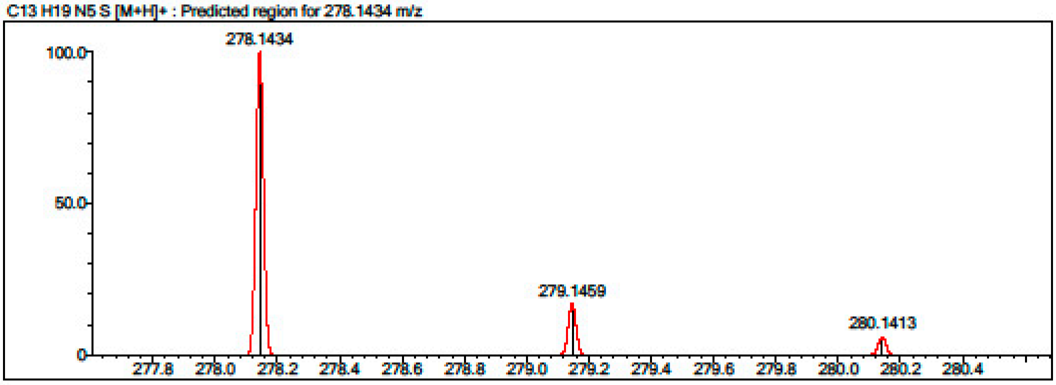

Rank	Score	Formula (M)	Ion	Meas. m/z	Pred. m/z	Df. (mDa)	Df. (ppm)	Iso	DBE
1	78.43	C13 H19 N5 S	[M+H]+	278.1426	278.1434	-0.8	-2.88	82.30	7.0

Figure S6. HRMS spectra of compound **2**

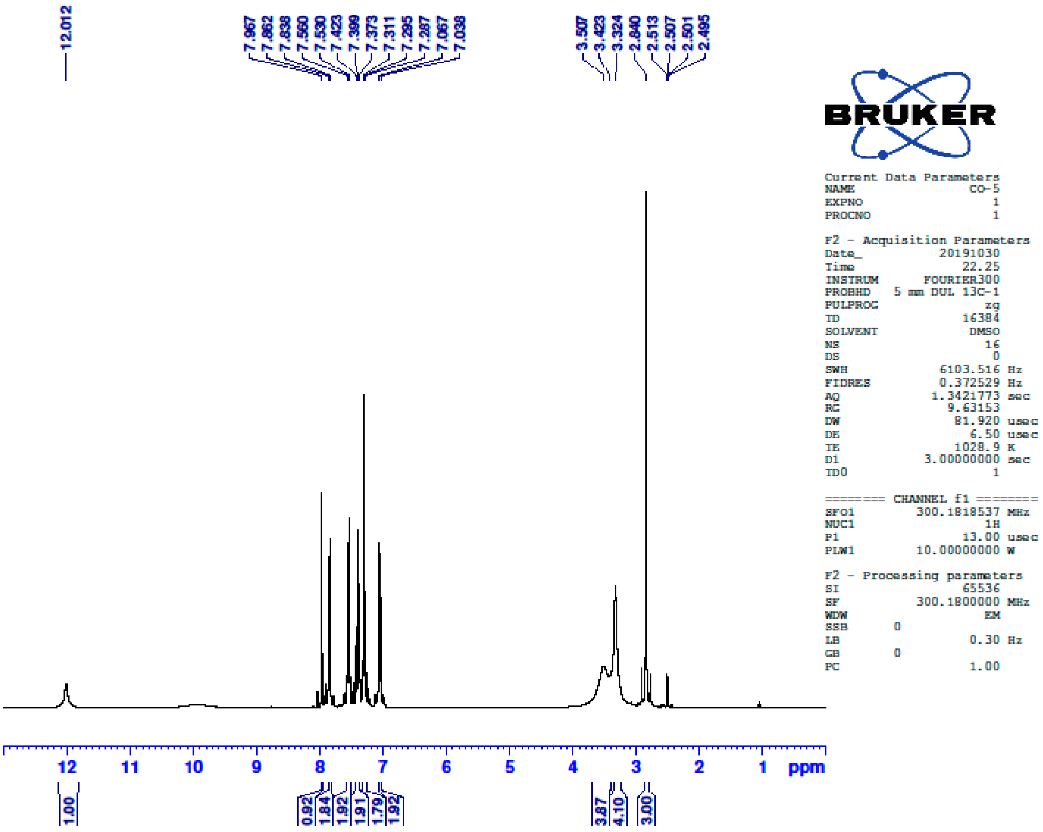

Figure S7. ^{1}H-NMR spectra of compound **3a**

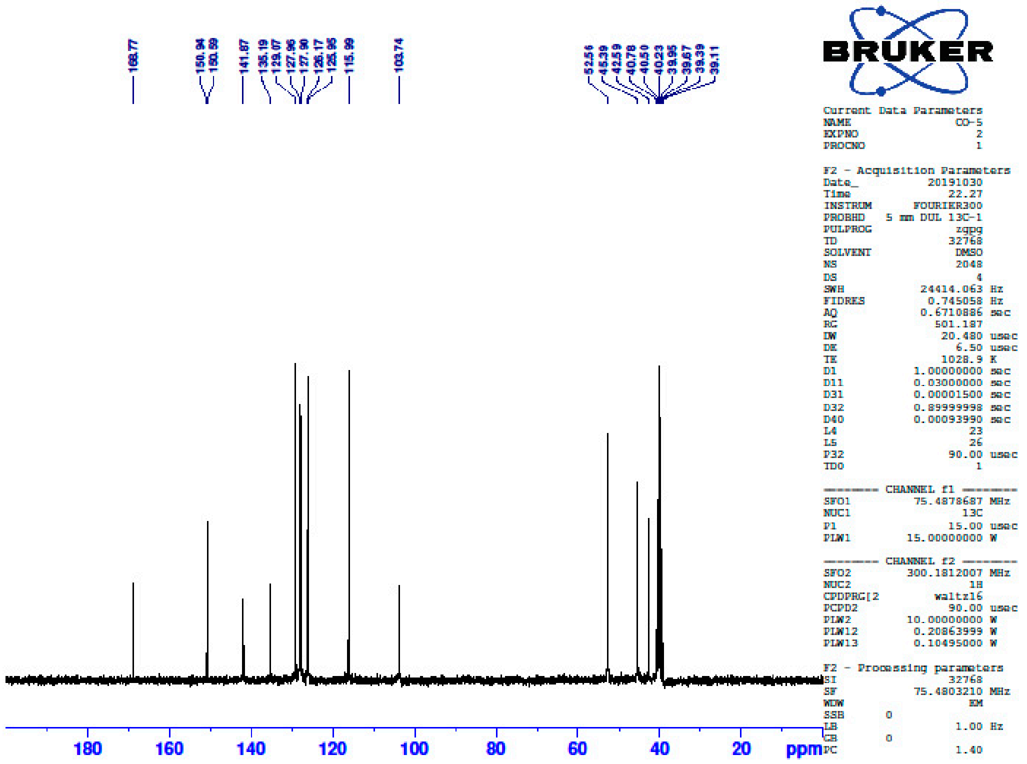

Figure S8. ^{13}C-NMR spectra of compound **3a**

Formula Predictor Report - CO-5_5.lcd Page 1 of 1

Data File: C:\LabSolutions\Data\Analiz\derya\CO-5_5.lcd

Elmt	Val.	Min	Max	Elmt	Val.	Min	Max	Elmt	Val.	Min	Max	Elmt	Val.	Min	Max	Use Adduct
H	1	0	40	O	2	0	2	S	2	1	1	Ru	2	0	0	H
C	4	21	35	F	1	0	1	Cl	1	0	2	Pd	2	0	0	
N	3	5	6	P	3	0	0	Br	1	0	1	I	3	0	0	

Error Margin (ppm): 25
HC Ratio: unlimited
Max Isotopes: 3
MSn Iso RI (%): 10.00

DBE Range: 10.0 - 20.0
Apply N Rule: yes
Isotope RI (%): 1.00
MSn Logic Mode: AND

Electron Ions: both
Use MSn Info: yes
Isotope Res: 9000
Max Results: 100

Event#: 1 MS(E+) Ret. Time : 2.987 Scan# : 449

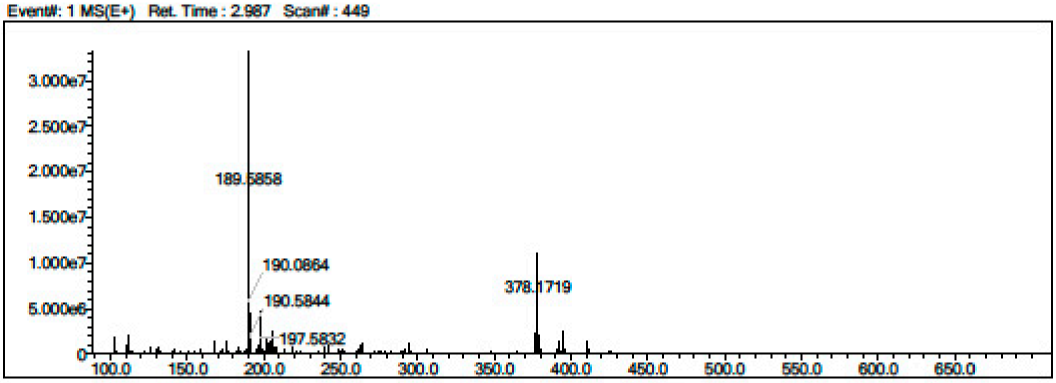

Measured region for 378.1719 m/z

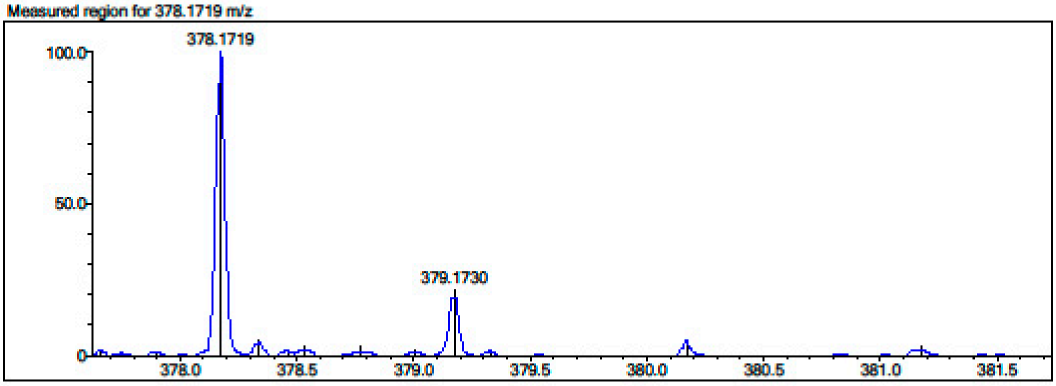

C21 H23 N5 S [M+H]+ : Predicted region for 378.1747 m/z

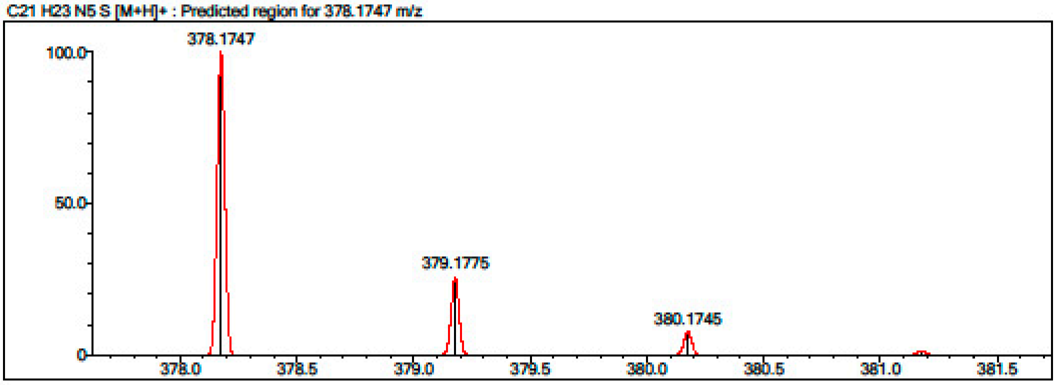

Rank	Score	Formula (M)	Ion	Meas. m/z	Pred. m/z	Df. (mDa)	Df. (ppm)	Iso	DBE
1	44.59	C21 H23 N5 S	[M+H]+	378.1719	378.1747	-2.8	-7.40	67.56	13.0

Figure S9. HRMS spectra of compound **3a**

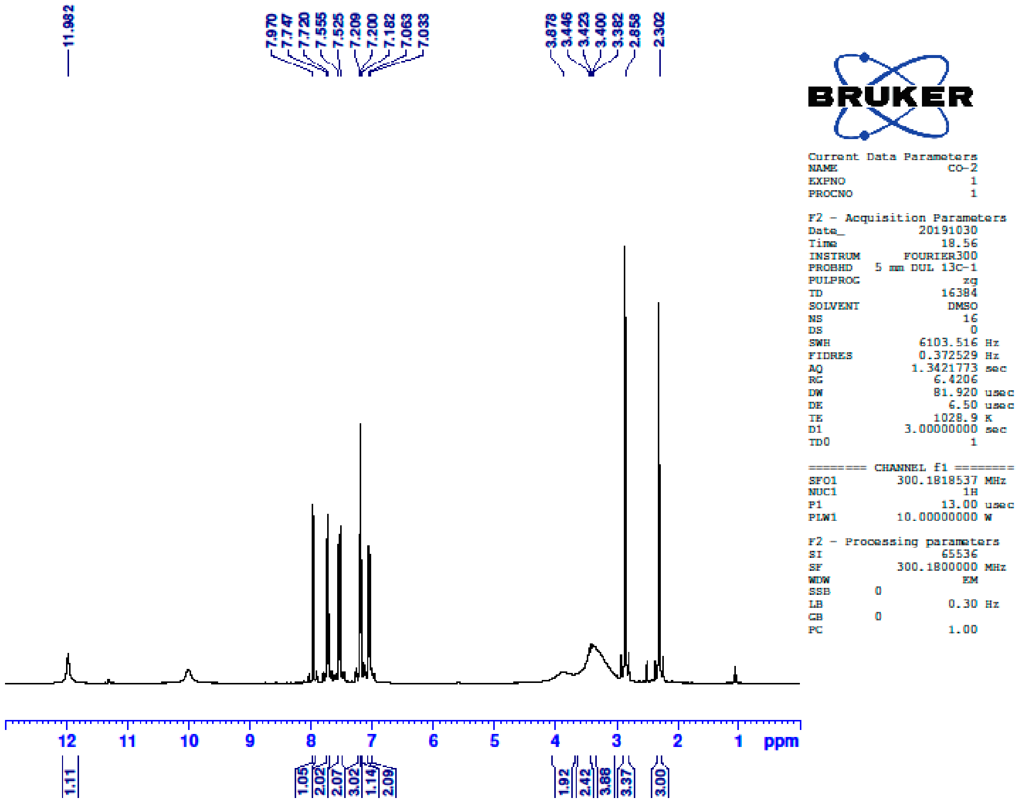

Figure S10. ^{1}H-NMR spectra of compound **3b**

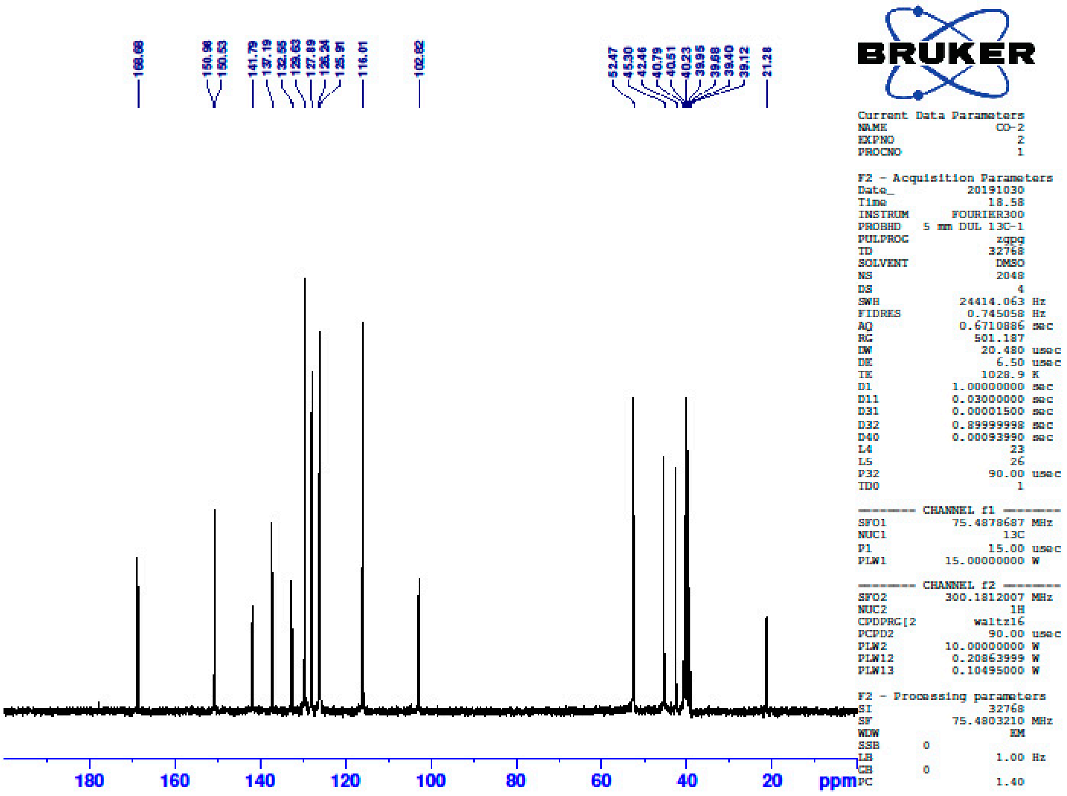

Figure S11. ^{13}C-NMR spectra of compound **3b**

Formula Predictor Report - CO-2_2.lcd Page 1 of 1

Data File: C:\LabSolutions\Data\Analiz\derya\CO-2_2.lcd

Elmt	Val.	Min	Max	Elmt	Val.	Min	Max	Elmt	Val.	Min	Max	Elmt	Val.	Min	Max	Use Adduct
H	1	0	40	O	2	0	1	S	2	1	1	Ru	2	0	0	H
C	4	21	35	F	1	0	0	Cl	1	0	2	Pd	2	0	0	
N	3	5	6	P	3	0	0	Br	1	0	1	I	3	0	0	

Error Margin (ppm):	25	DBE Range:	10.0 - 20.0	Electron Ions:	both
HC Ratio:	unlimited	Apply N Rule:	yes	Use MSn Info:	yes
Max Isotopes:	3	Isotope RI (%):	1.00	Isotope Res:	9000
MSn Iso RI (%):	10.00	MSn Logic Mode:	AND	Max Results:	100

Event#: 1 MS(E+) Ret. Time : 3.200 Scan# : 481

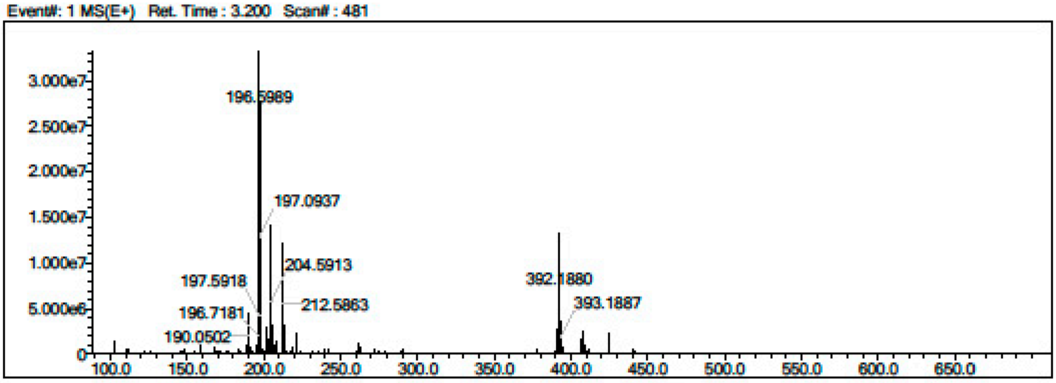

Measured region for 392.1880 m/z

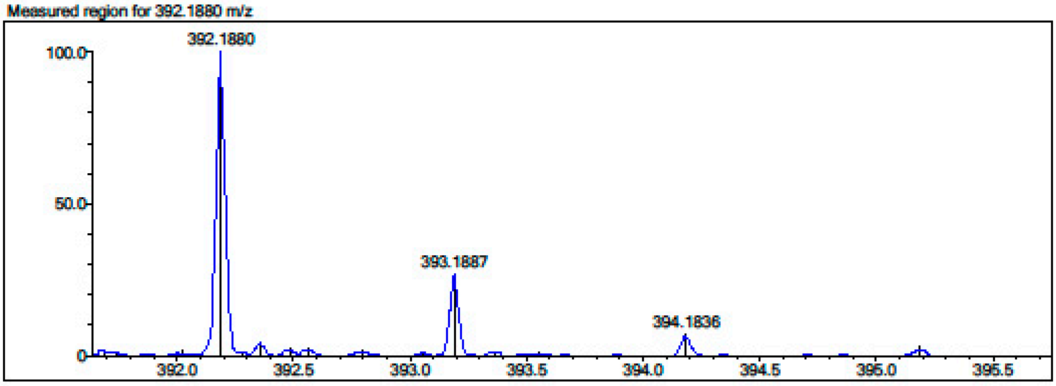

C22 H25 N5 S [M+H]+ : Predicted region for 392.1903 m/z

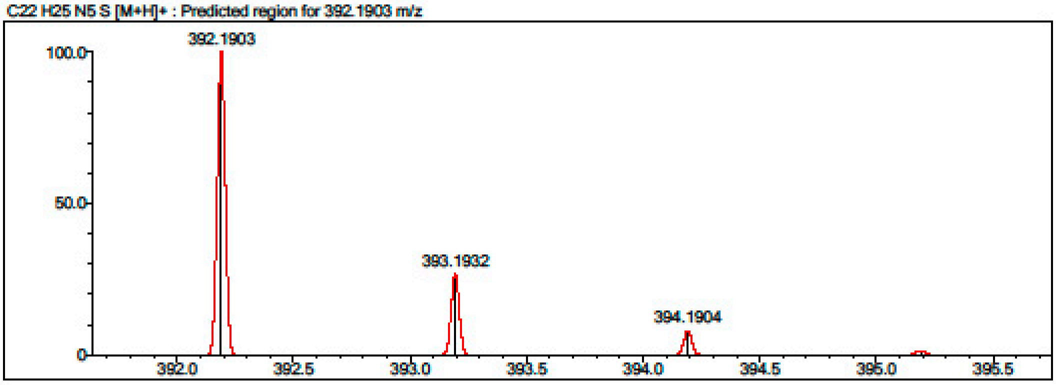

Rank	Score	Formula (M)	Ion	Meas. m/z	Pred. m/z	Df. (mDa)	Df. (ppm)	Iso	DBE
1	54.22	C22 H25 N5 S	[M+H]+	392.1880	392.1903	-2.3	-5.86	66.61	13.0

Figure S12. HRMS spectra of compound **3b**

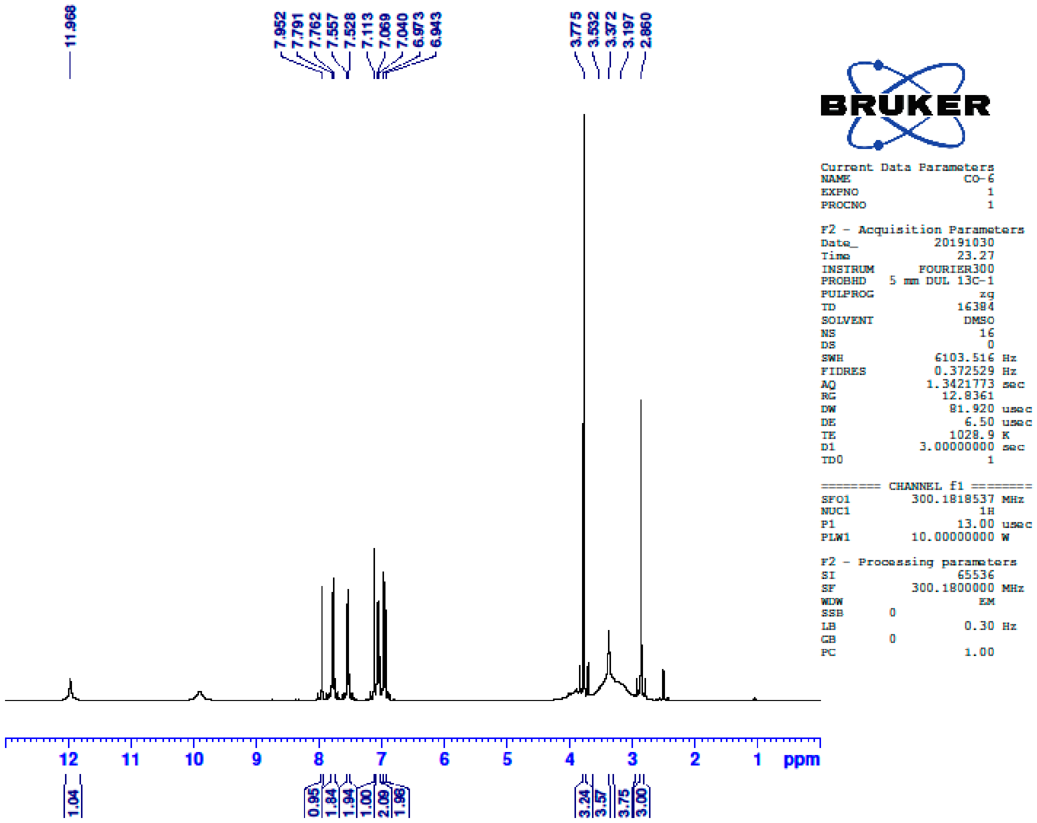

Figure S13. ^{1}H-NMR spectra of compound **3c**

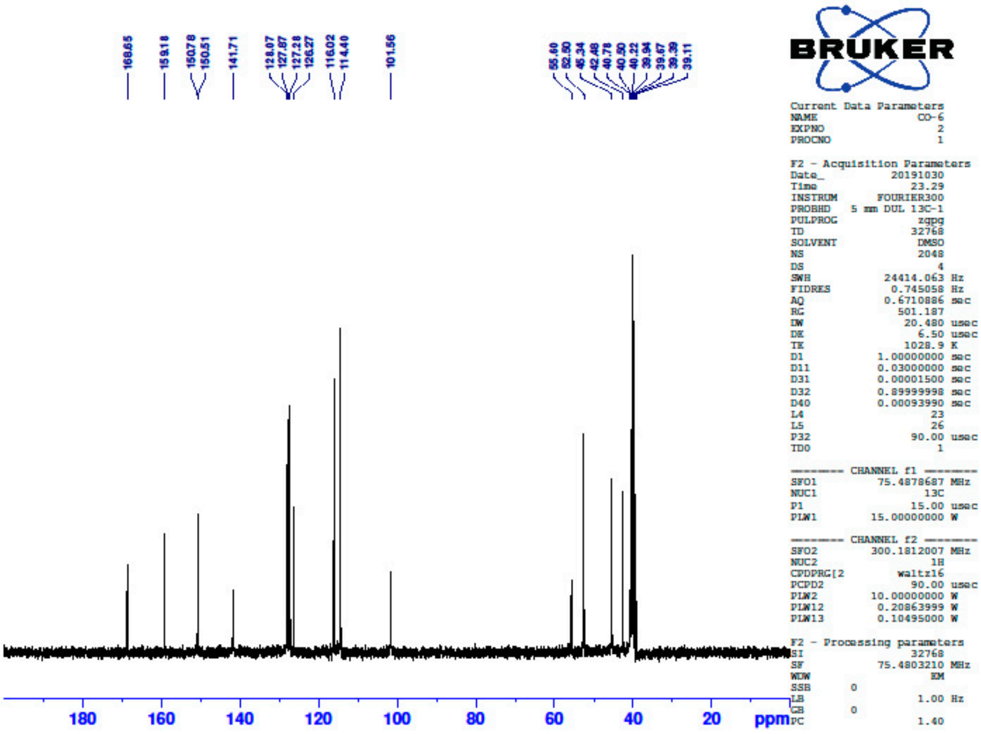

Figure S14. ^{13}C-NMR spectra of compound **3c**

Formula Predictor Report - CO-6_6.lcd

Data File: C:\LabSolutions\Data\Analiz\derya\CO-6_6.lcd

Elmt	Val.	Min	Max	Elmt	Val.	Min	Max	Elmt	Val.	Min	Max	Elmt	Val.	Min	Max	Use Adduct
H	1	0	40	O	2	0	2	S	2	1	1	Ru	2	0	0	H
C	4	21	35	F	1	0	1	Cl	1	0	2	Pd	2	0	0	
N	3	5	6	P	3	0	0	Br	1	0	1	I	3	0	0	

Error Margin (ppm):	25	DBE Range:	10.0 - 20.0	Electron Ions:	both
HC Ratio:	unlimited	Apply N Rule:	yes	Use MSn Info:	yes
Max Isotopes:	3	Isotope RI (%):	1.00	Isotope Res:	9000
MSn Iso RI (%):	10.00	MSn Logic Mode:	AND	Max Results:	100

Event#: 1 MS(E+) Ret. Time : 2.640 Scan# : 397

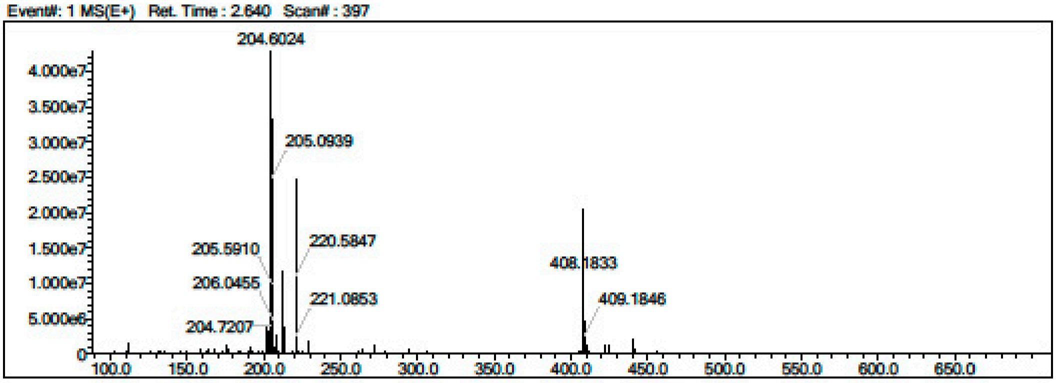

Measured region for 408.1833 m/z

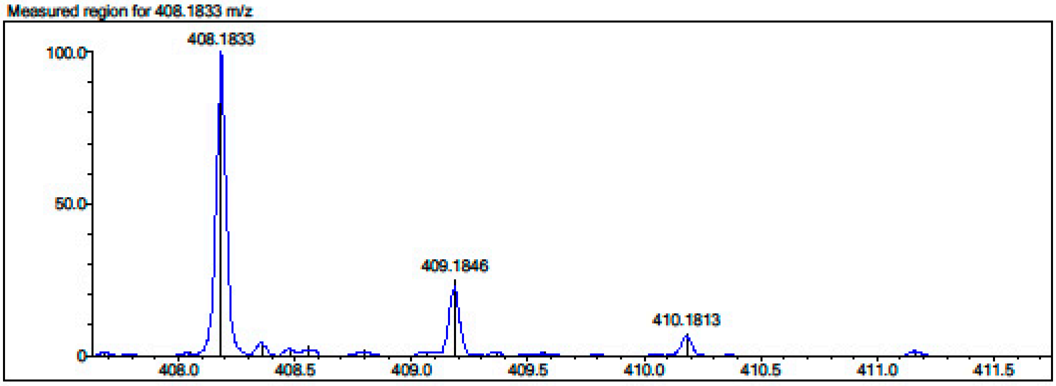

C22 H25 N5 O S [M+H]+ : Predicted region for 408.1853 m/z

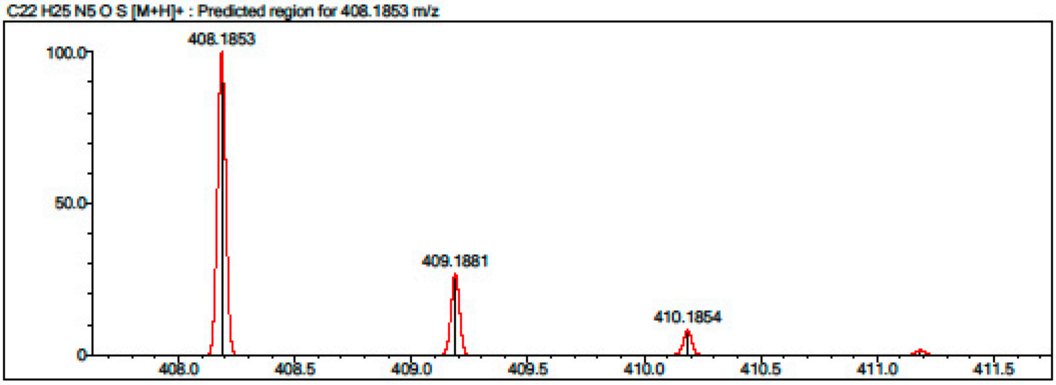

Rank	Score	Formula (M)	Ion	Meas. m/z	Pred. m/z	Df. (mDa)	Df. (ppm)	Iso	DBE
1	56.93	C22 H25 N5 O S	[M+H]+	408.1833	408.1853	-2.0	-4.90	63.08	13.0

Figure S15. HRMS spectra of compound **3c**

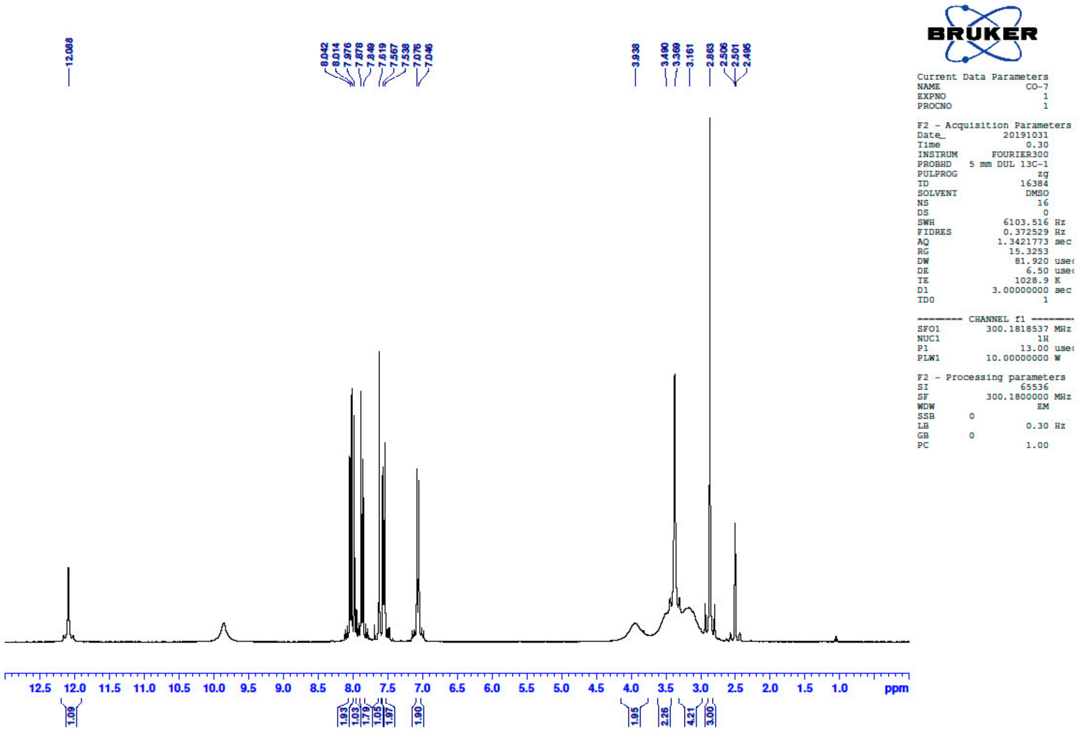

Figure S16. ^{1}H-NMR spectra of compound **3d**

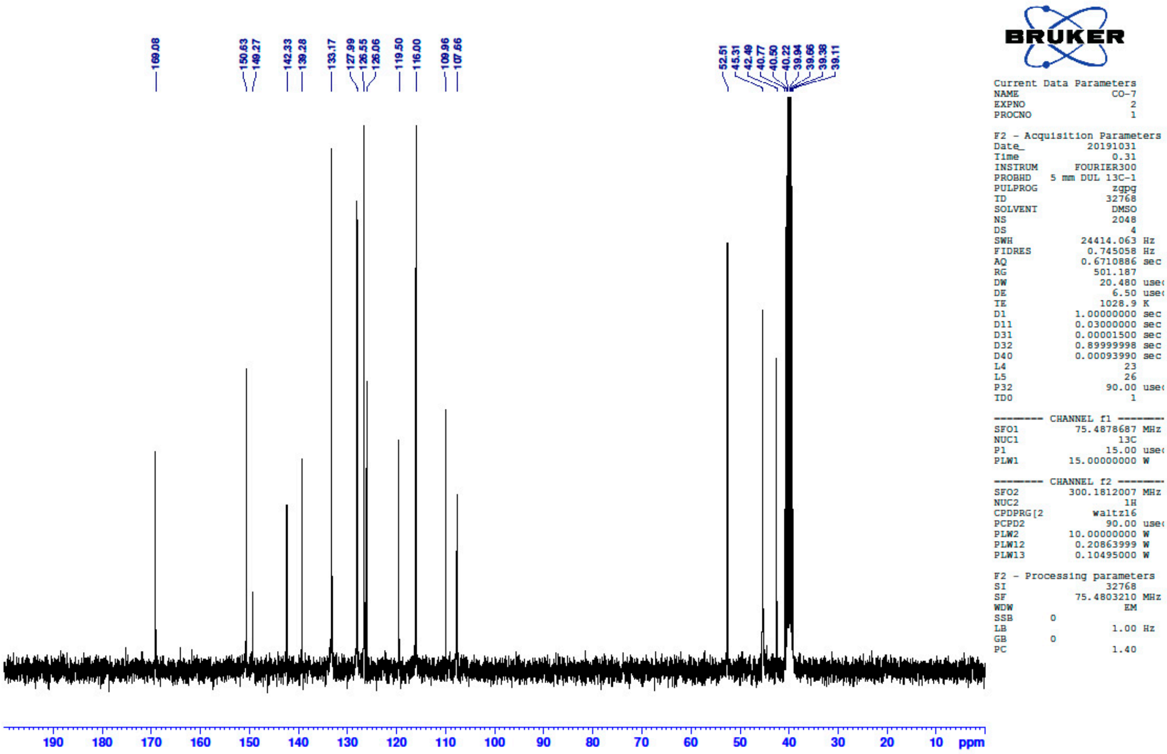

Figure S17. ^{13}C-NMR spectra of compound **3d**

Formula Predictor Report - CO-7_7.lcd Page 1 of 1

Data File: C:\LabSolutions\Data\Analiz\derya\CO-7_7.lcd

Elmt	Val.	Min	Max	Elmt	Val.	Min	Max	Elmt	Val.	Min	Max	Elmt	Val.	Min	Max	Use Adduct
H	1	0	40	O	2	0	2	S	2	1	1	Ru	2	0	0	H
C	4	21	35	F	1	0	1	Cl	1	0	2	Pd	2	0	0	
N	3	5	6	P	3	0	0	Br	1	0	1	I	3	0	0	

Error Margin (ppm): 25
HC Ratio: unlimited
Max Isotopes: 3
MSn Iso RI (%): 10.00

DBE Range: 10.0 - 20.0
Apply N Rule: yes
Isotope RI (%): 1.00
MSn Logic Mode: AND

Electron Ions: both
Use MSn Info: yes
Isotope Res: 9000
Max Results: 100

Event#: 1 MS(E+) Ret. Time : 2.453 Scan# : 369

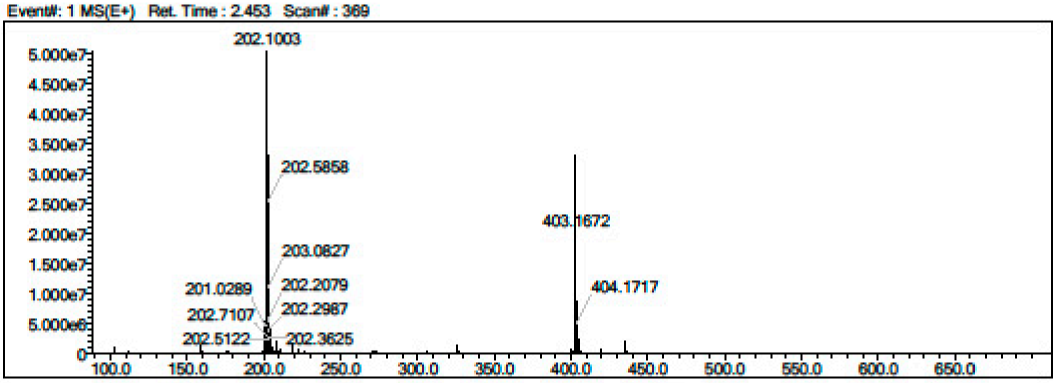

Measured region for 403.1672 m/z

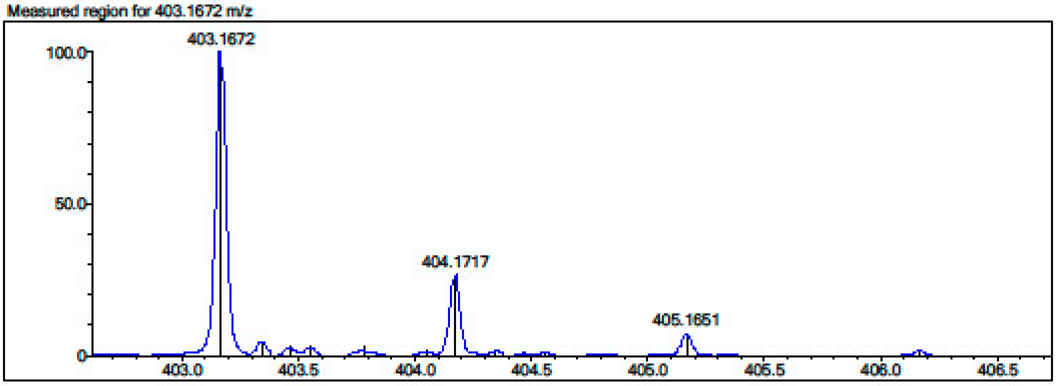

C22 H22 N6 S [M+H]+ : Predicted region for 403.1699 m/z

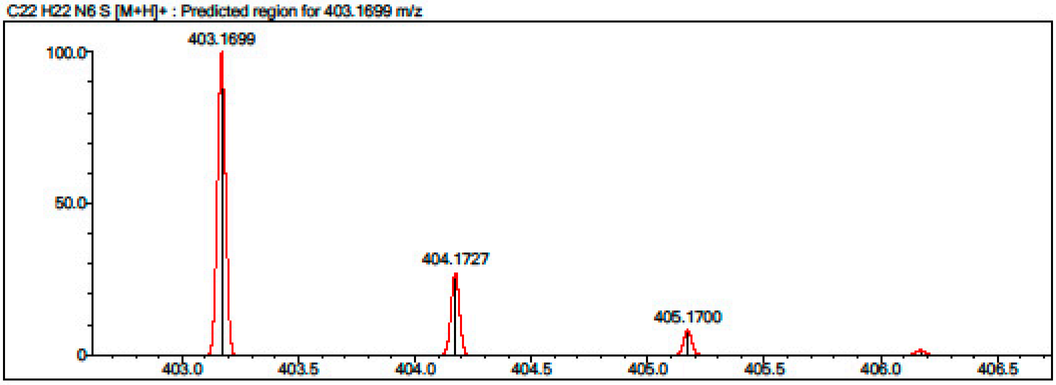

Rank	Score	Formula (M)	Ion	Meas. m/z	Pred. m/z	Df. (mDa)	Df. (ppm)	Iso	DBE
1	60.74	C22 H22 N6 S	[M+H]+	403.1672	403.1699	-2.7	-6.70	83.21	15.0

Figure S18. HRMS spectra of compound **3d**

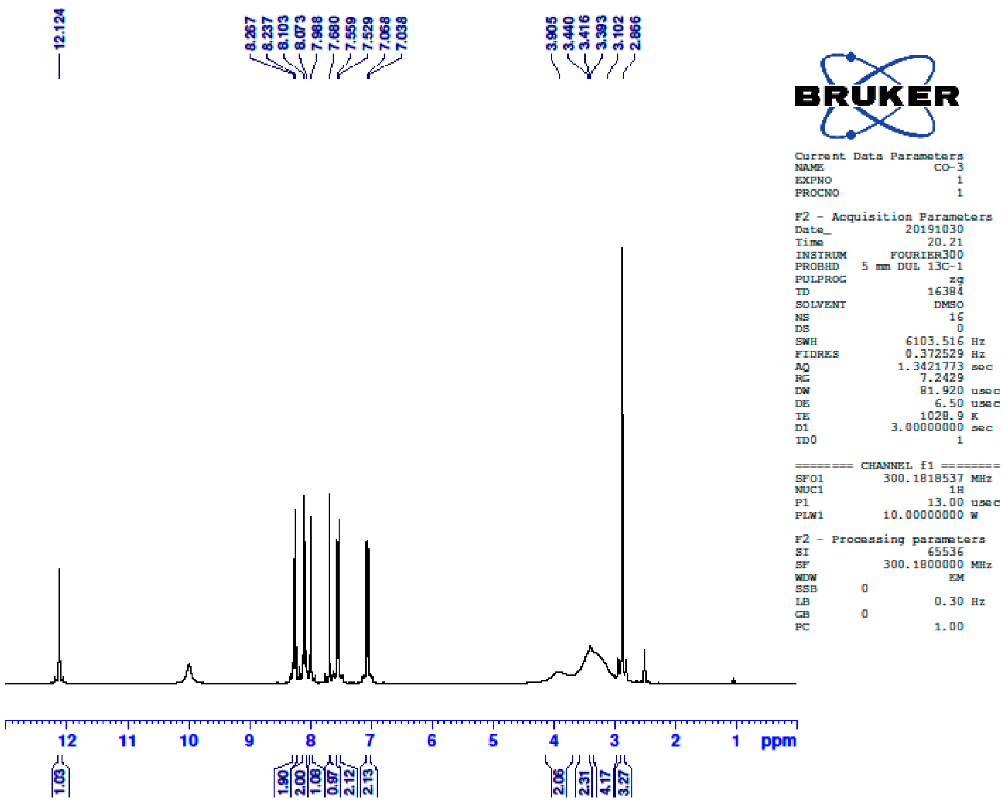

Figure S19. ^{1}H-NMR spectra of compound **3e**

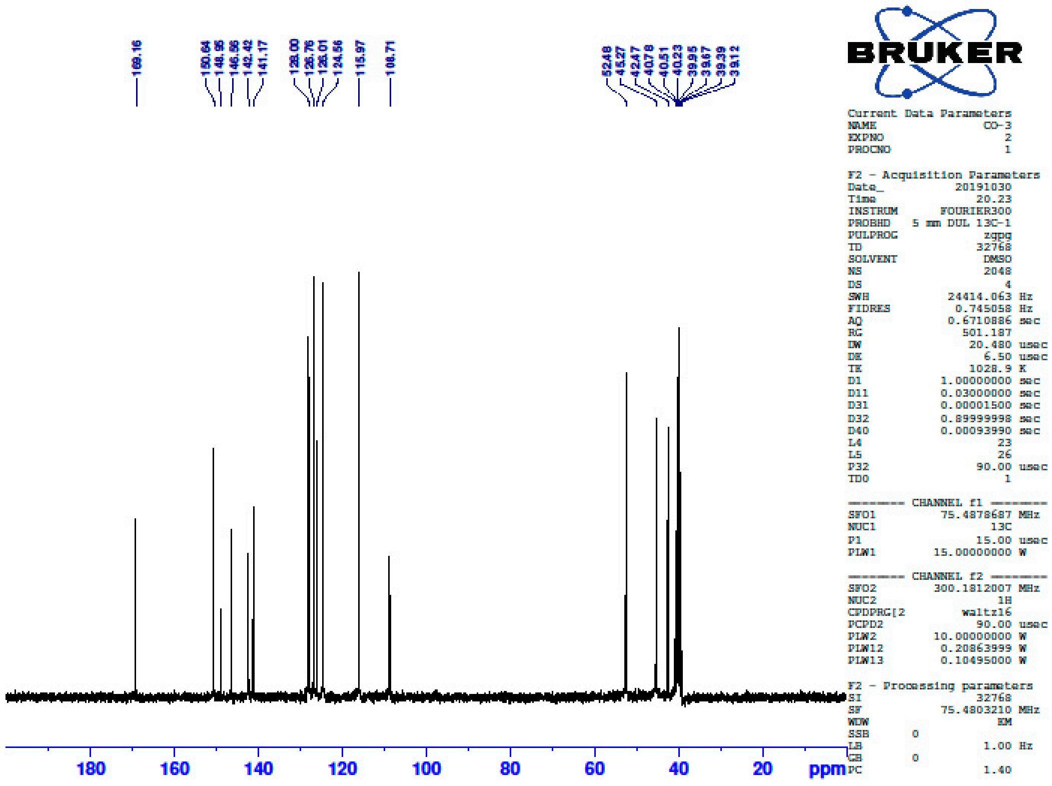

Figure S20. ^{13}C-NMR spectra of compound **3e**

Formula Predictor Report - CO-3_3.lcd Page 1 of 1

Data File: C:\LabSolutions\Data\Analiz\derya\CO-3_3.lcd

Elmt	Val.	Min	Max	Elmt	Val.	Min	Max	Elmt	Val.	Min	Max	Elmt	Val.	Min	Max	Use Adduct
H	1	0	40	O	2	0	2	S	2	1	1	Ru	2	0	0	H
C	4	21	35	F	1	0	0	Cl	1	0	2	Pd	2	0	0	
N	3	5	6	P	3	0	0	Br	1	0	1	I	3	0	0	

Error Margin (ppm):	25	DBE Range:	10.0 - 20.0	Electron Ions:	both
HC Ratio:	unlimited	Apply N Rule:	yes	Use MSn Info:	yes
Max Isotopes:	3	Isotope RI (%):	1.00	Isotope Res:	9000
MSn Iso RI (%):	10.00	MSn Logic Mode:	AND	Max Results:	100

Event#: 1 MS(E+) Ret. Time : 3.027 Scan# : 455

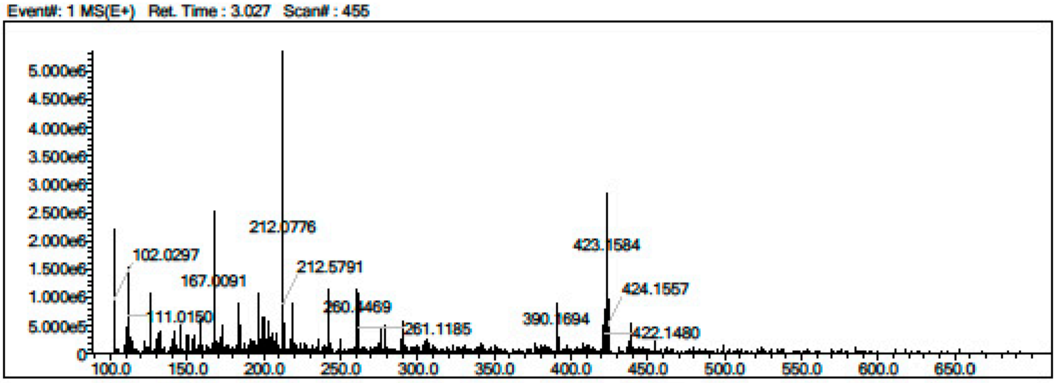

Measured region for 423.1584 m/z

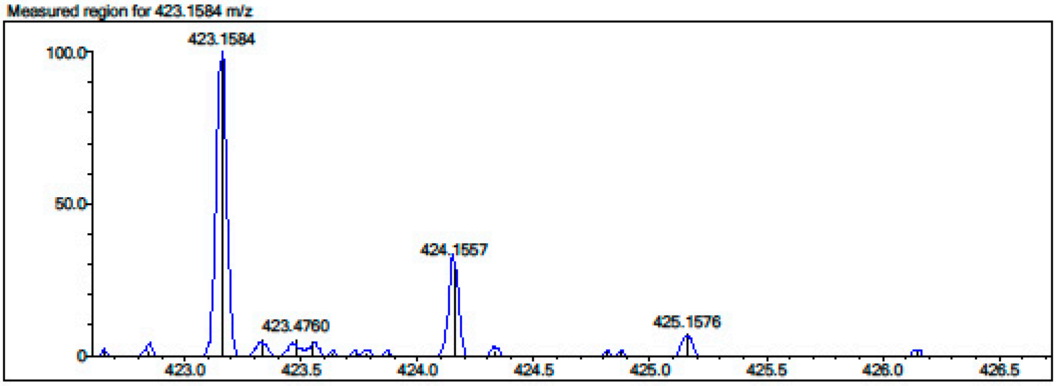

C21 H22 N6 O2 S [M+H]+ : Predicted region for 423.1598 m/z

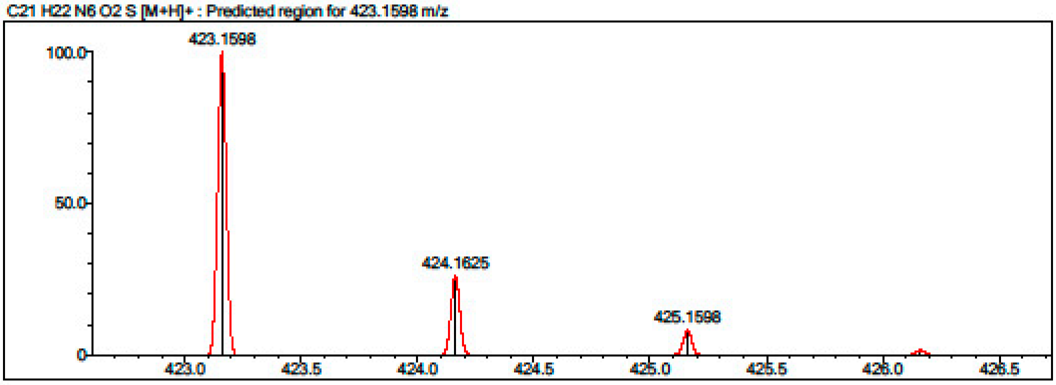

Rank	Score	Formula (M)	Ion	Meas. m/z	Pred. m/z	Df. (mDa)	Df. (ppm)	Iso	DBE
1	71.93	C21 H22 N6 O2 S	[M+H]+	423.1584	423.1598	-1.4	-3.31	76.34	14.0

Figure S21. HRMS spectra of compound **3e**

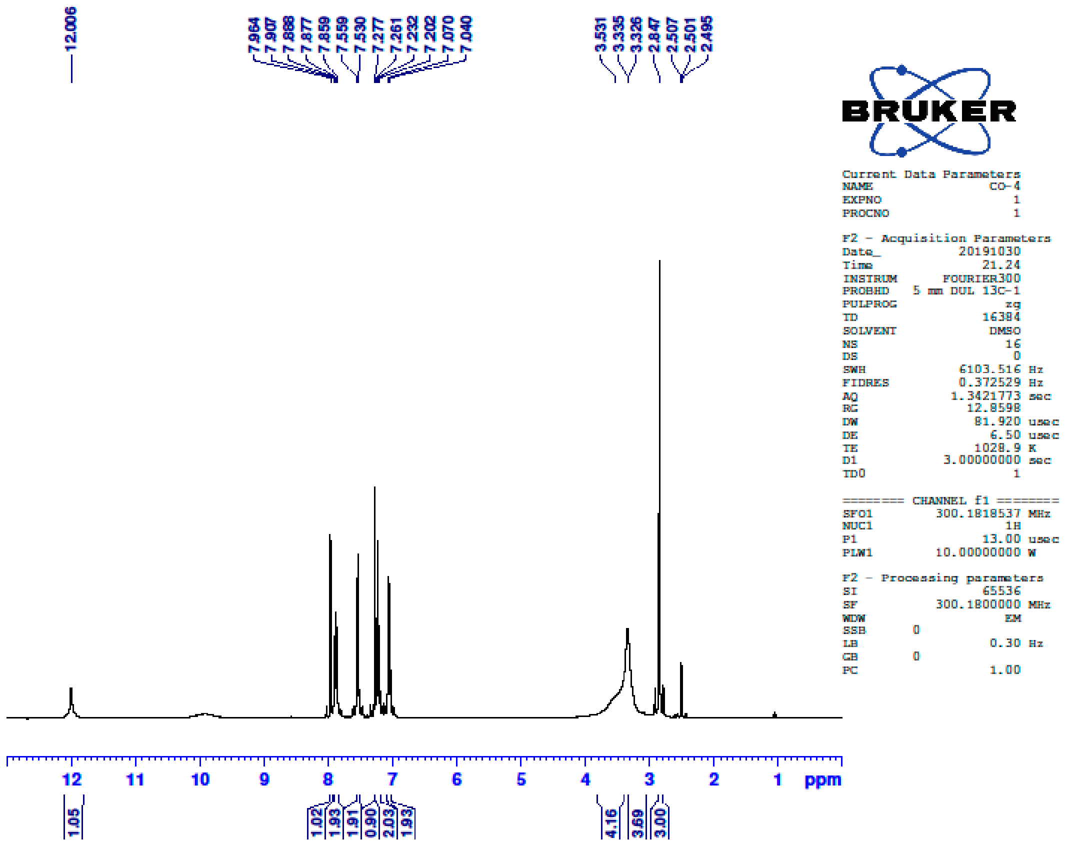

Figure S22. ^{1}H-NMR spectra of compound **3f**

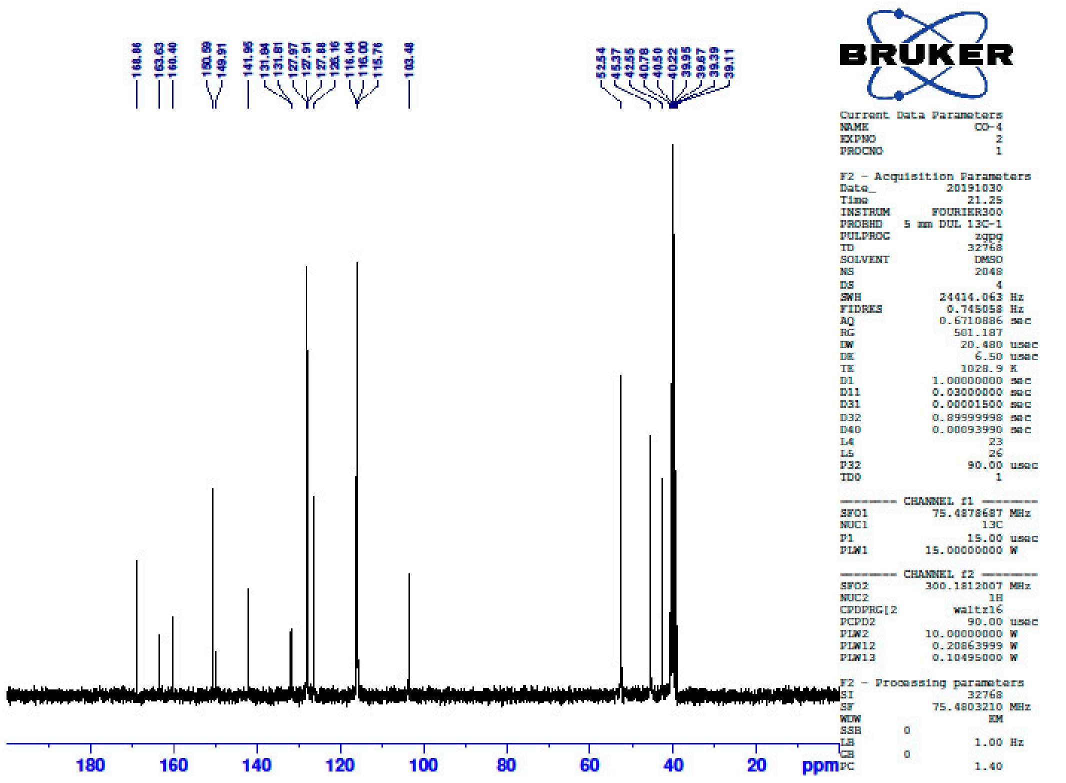

Figure S23. ^{13}C-NMR spectra of compound **3f**

Formula Predictor Report - CO-4_4.lcd   Page 1 of 1

Data File: C:\LabSolutions\Data\Analiz\derya\CO-4_4.lcd

Elmt	Val.	Min	Max	Elmt	Val.	Min	Max	Elmt	Val.	Min	Max	Elmt	Val.	Min	Max	Use Adduct
H	1	0	40	O	2	0	2	S	2	1	1	Ru	2	0	0	H
C	4	21	35	F	1	0	1	Cl	1	0	2	Pd	2	0	0	
N	3	5	6	P	3	0	0	Br	1	0	1	I	3	0	0	

Error Margin (ppm):	25	DBE Range:	10.0 - 20.0	Electron Ions:	both
HC Ratio:	unlimited	Apply N Rule:	yes	Use MSn Info:	yes
Max Isotopes:	3	Isotope RI (%):	1.00	Isotope Res:	9000
MSn Iso RI (%):	10.00	MSn Logic Mode:	AND	Max Results:	100

Event#: 1 MS(E+) Ret. Time : 2.613 Scan# : 393

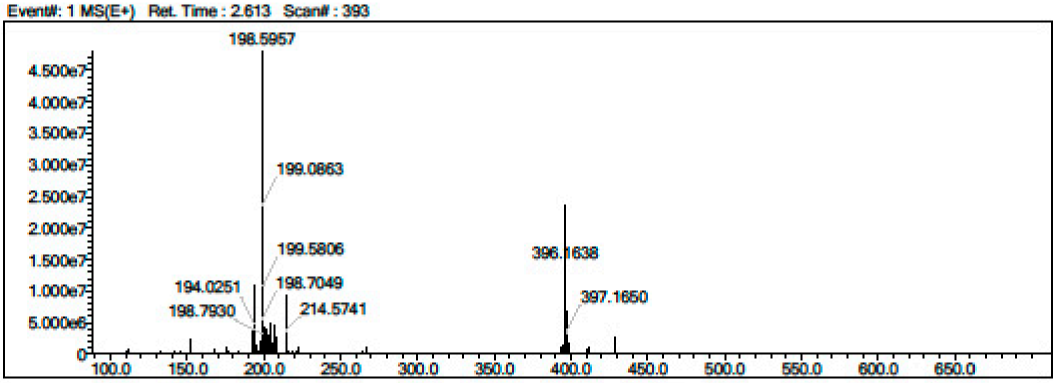

Measured region for 396.1638 m/z

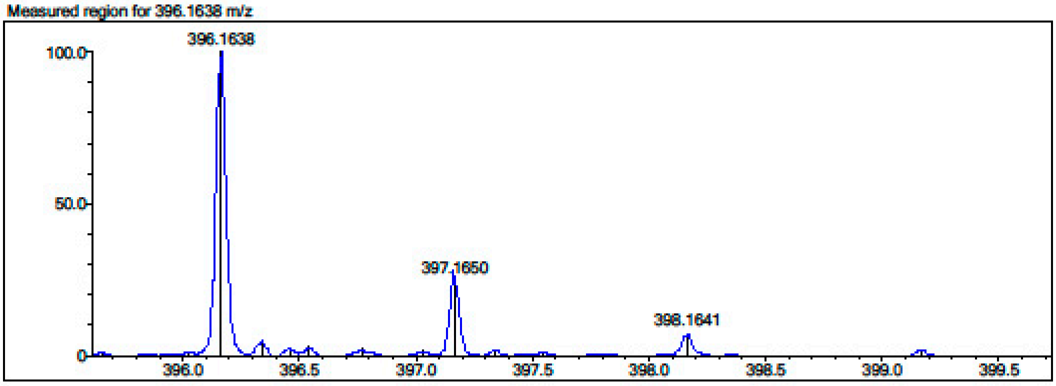

C21 H22 N5 F S [M+H]+ : Predicted region for 396.1653 m/z

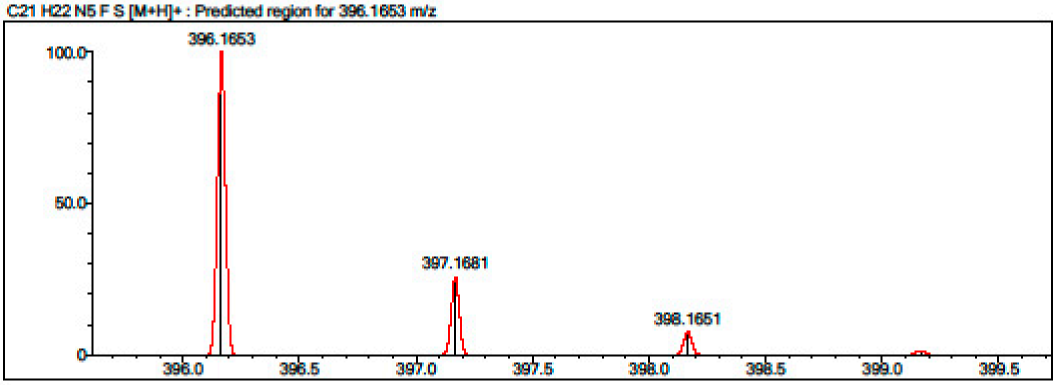

Rank	Score	Formula (M)	Ion	Meas. m/z	Pred. m/z	Df. (mDa)	Df. (ppm)	Iso	DBE
1	91.60	C21 H22 N5 F S	[M+H]+	396.1638	396.1653	-1.5	-3.79	98.47	13.0

Figure S24. HRMS spectra of compound **3f**

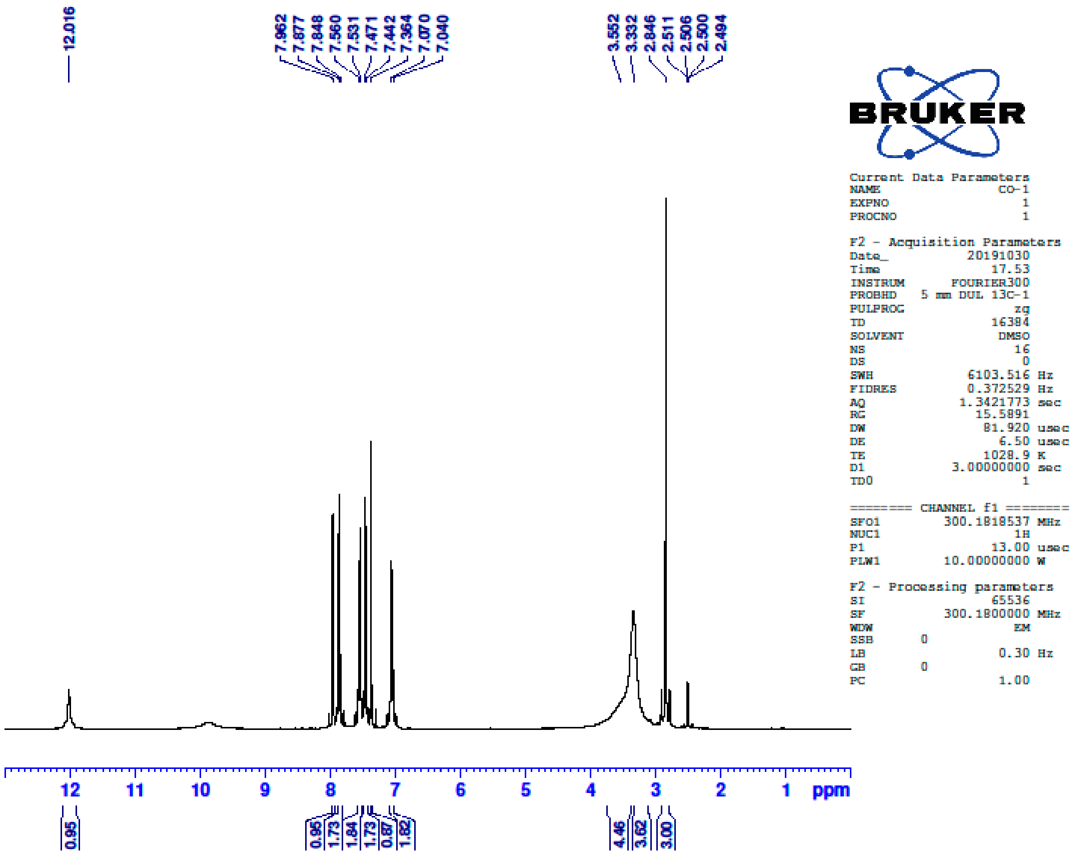

Figure S25. ^{1}H-NMR spectra of compound **3g**

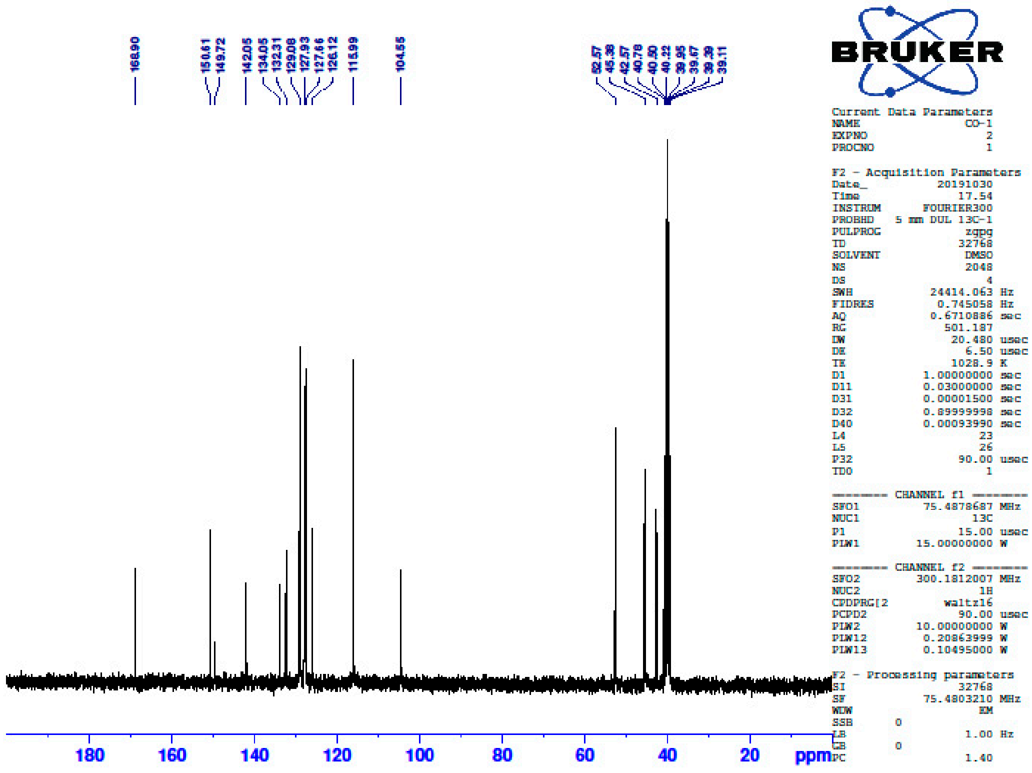

Figure S26. ^{13}C-NMR spectra of compound **3g**

Data File: C:\LabSolutions\Data\Analiz\derya\CO-1_1.lcd

Elmt	Val.	Min	Max	Elmt	Val.	Min	Max	Elmt	Val.	Min	Max	Elmt	Val.	Min	Max	Use Adduct
H	1	0	40	O	2	0	1	S	2	1	1	Ru	2	0	0	H
C	4	21	35	F	1	0	0	Cl	1	0	2	Pd	2	0	0	
N	3	5	6	P	3	0	0	Br	1	0	1	I	3	0	0	

Error Margin (ppm):	25	DBE Range:	10.0 - 20.0	Electron Ions:	both
HC Ratio:	unlimited	Apply N Rule:	yes	Use MSn Info:	yes
Max Isotopes:	3	Isotope RI (%):	1.00	Isotope Res:	9000
MSn Iso RI (%):	10.00	MSn Logic Mode:	AND	Max Results:	100

Event#: 1 MS(E+) Ret. Time : 2.533 Scan# : 381

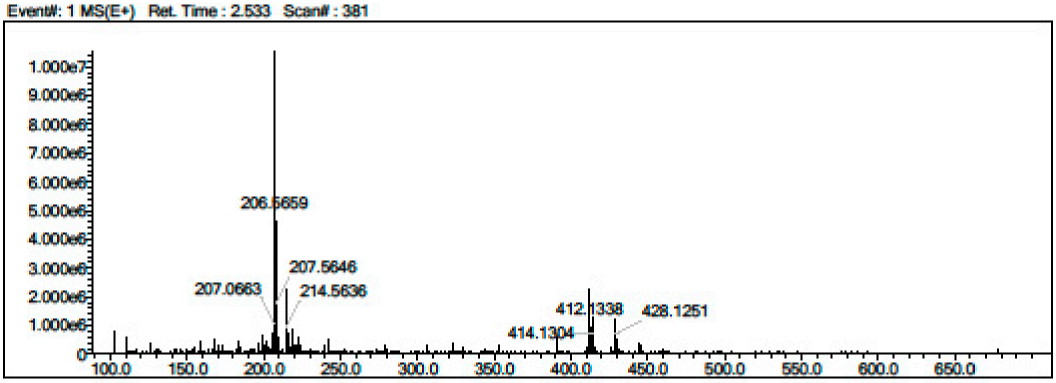

Measured region for 412.1338 m/z

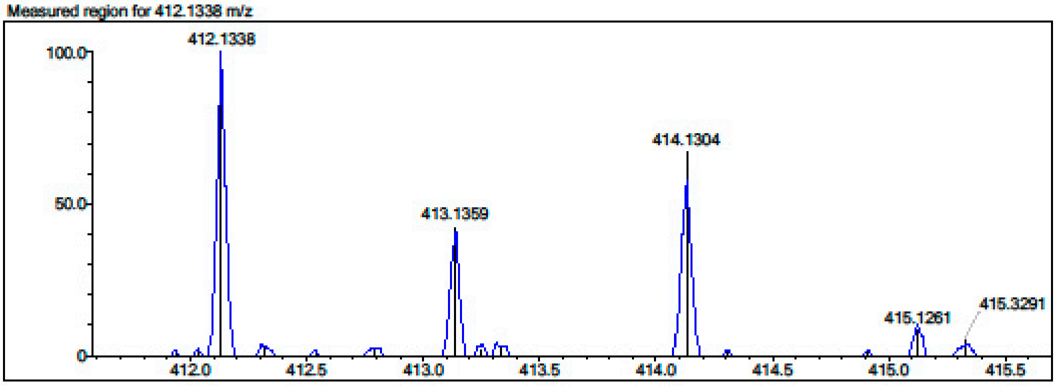

C21 H22 N5 S Cl [M+H]+ : Predicted region for 412.1357 m/z

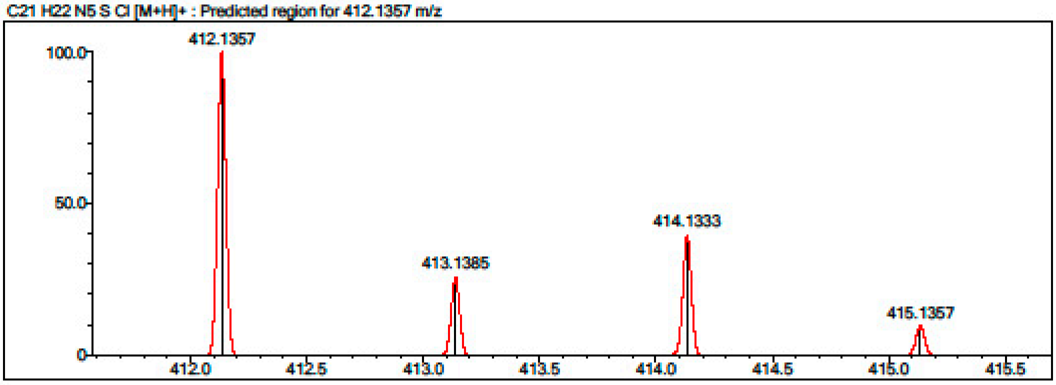

Rank	Score	Formula (M)	Ion	Meas. m/z	Pred. m/z	Df. (mDa)	Df. (ppm)	Iso	DBE
1	39.31	C21 H22 N5 S Cl	[M+H]+	412.1338	412.1357	-1.9	-4.61	43.21	13.0

Figure S27. HRMS spectra of compound **3g**

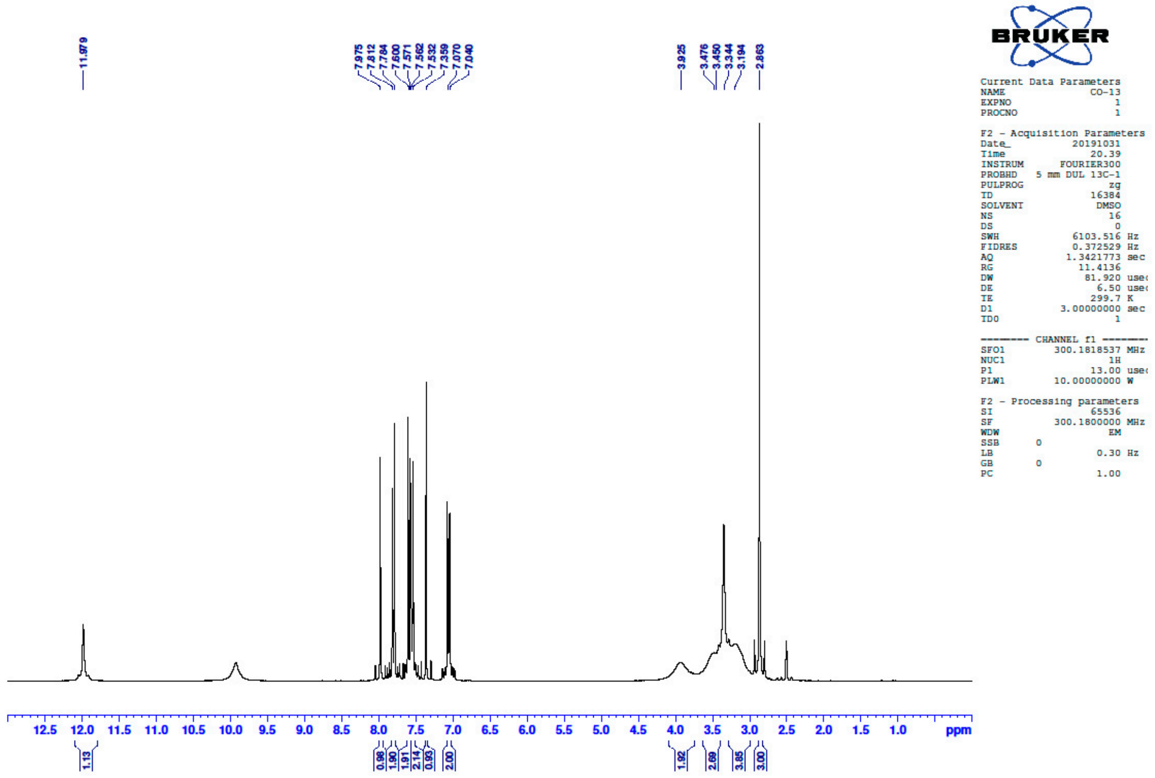

Figure S28. ¹H-NMR spectra of compound **3h**

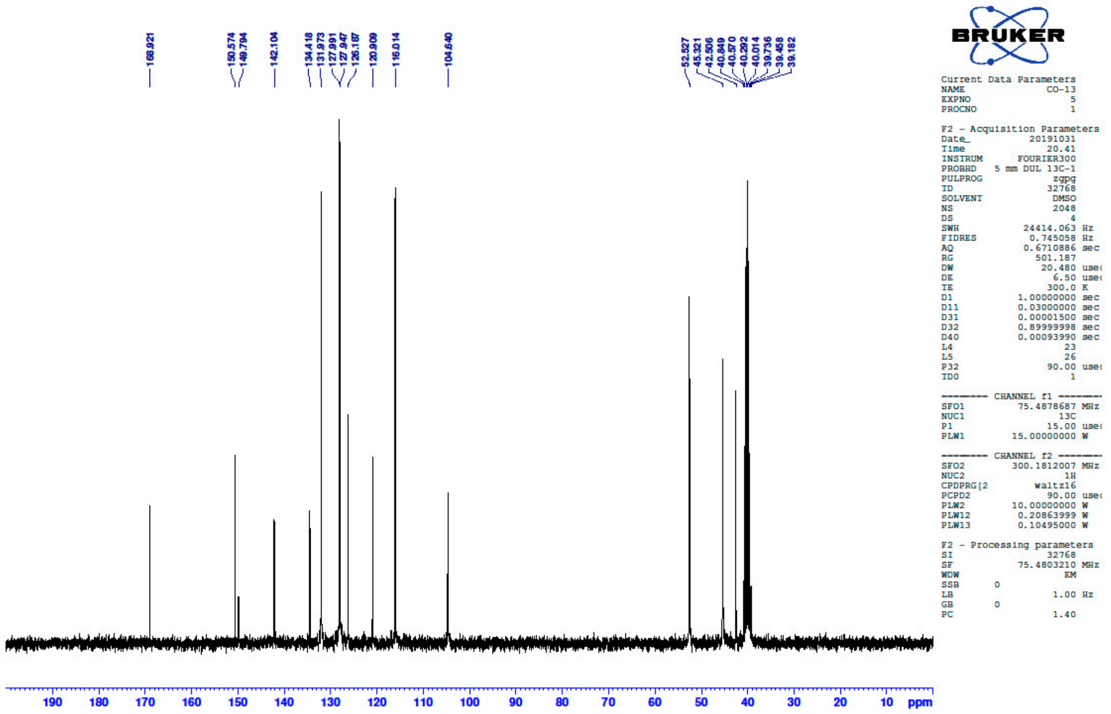

Figure S29. ¹³C-NMR spectra of compound **3h**

Formula Predictor Report - CO-13_12.lcd Page 1 of 1

Data File: C:\LabSolutions\Data\Analiz\derya\CO-13_12.lcd

Elmt	Val.	Min	Max	Elmt	Val.	Min	Max	Elmt	Val.	Min	Max	Elmt	Val.	Min	Max	Use Adduct
H	1	0	40	O	2	0	2	S	2	1	1	Ru	2	0	0	H
C	4	21	35	F	1	0	0	Cl	1	0	1	Pd	2	0	0	
N	3	5	6	P	3	0	0	Br	1	0	1	I	3	0	0	

Error Margin (ppm):	20	DBE Range:	10.0 - 20.0	Electron Ions:	both
HC Ratio:	unlimited	Apply N Rule:	yes	Use MSn Info:	yes
Max Isotopes:	3	Isotope RI (%):	1.00	Isotope Res:	9000
MSn Iso RI (%):	10.00	MSn Logic Mode:	AND	Max Results:	100

Event#: 1 MS(E+) Ret. Time : 3.533 Scan# : 531

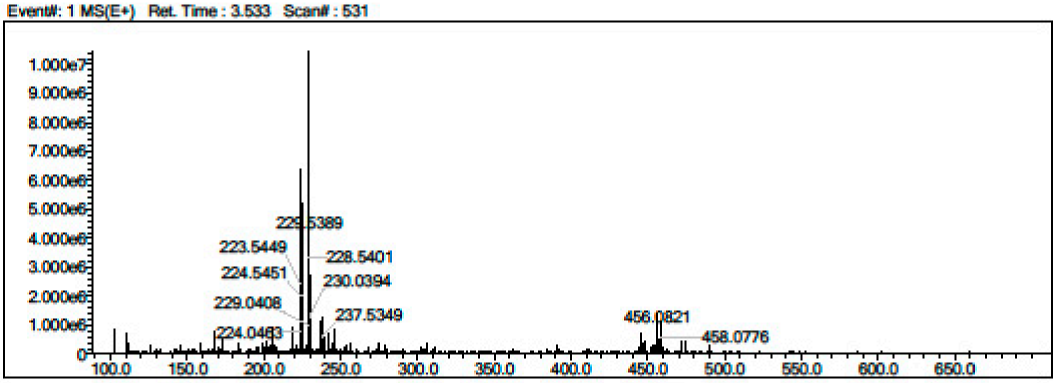

Measured region for 456.0821 m/z

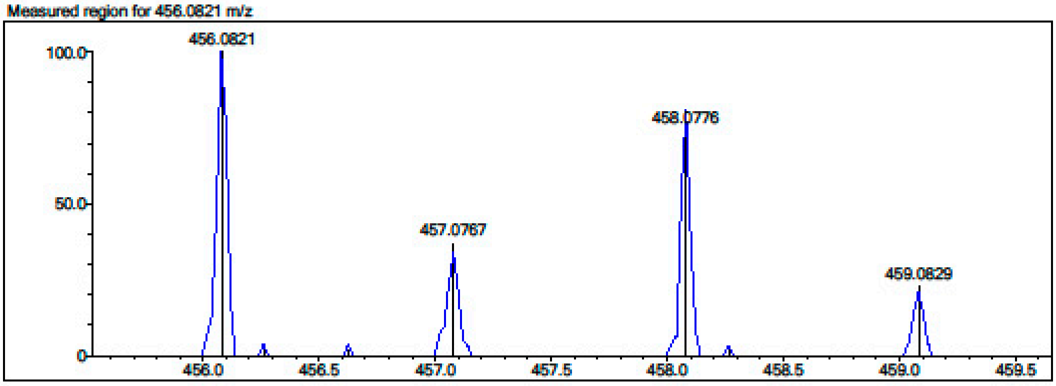

C21 H22 N5 S Br [M+H]+ : Predicted region for 456.0852 m/z

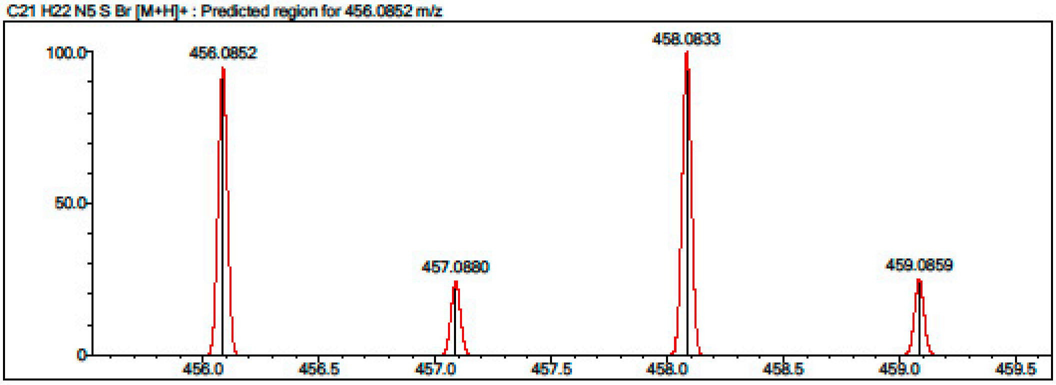

Rank	Score	Formula (M)	Ion	Meas. m/z	Pred. m/z	Df. (mDa)	Df. (ppm)	Iso	DBE
1	31.82	C21 H22 N5 S Br	[M+H]+	456.0821	456.0852	-3.1	-6.80	44.20	13.0

Figure S30. HRMS spectra of compound **3h**

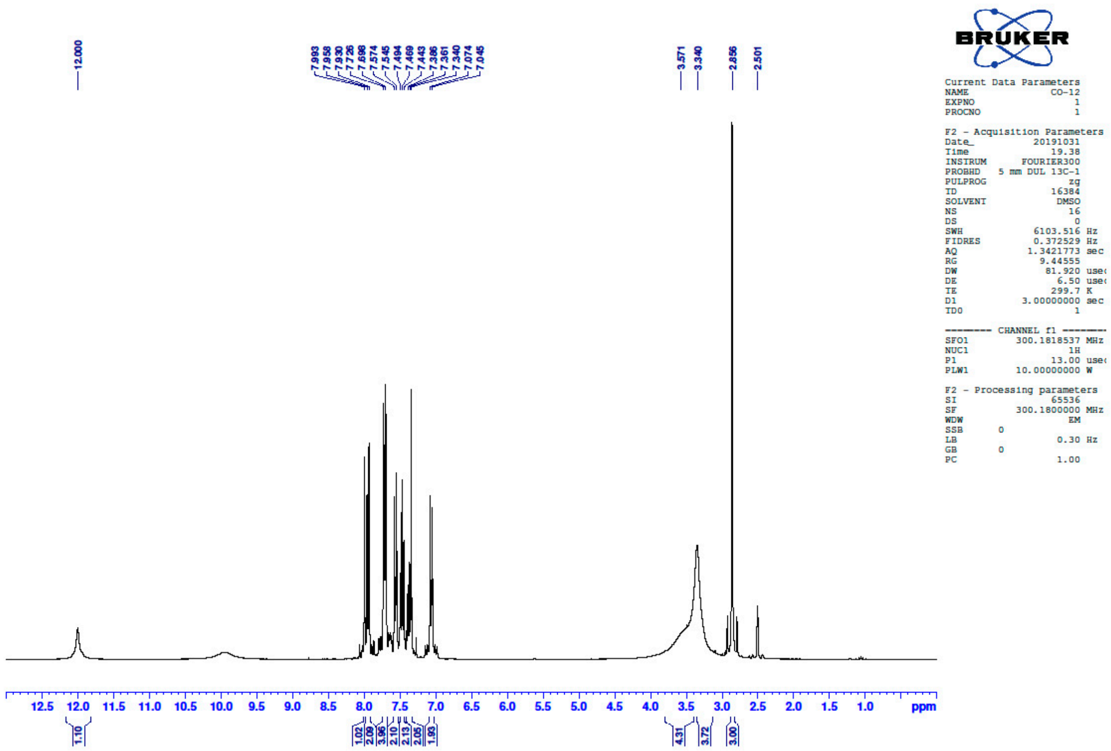

Figure S31. ^{1}H-NMR spectra of compound **3i**

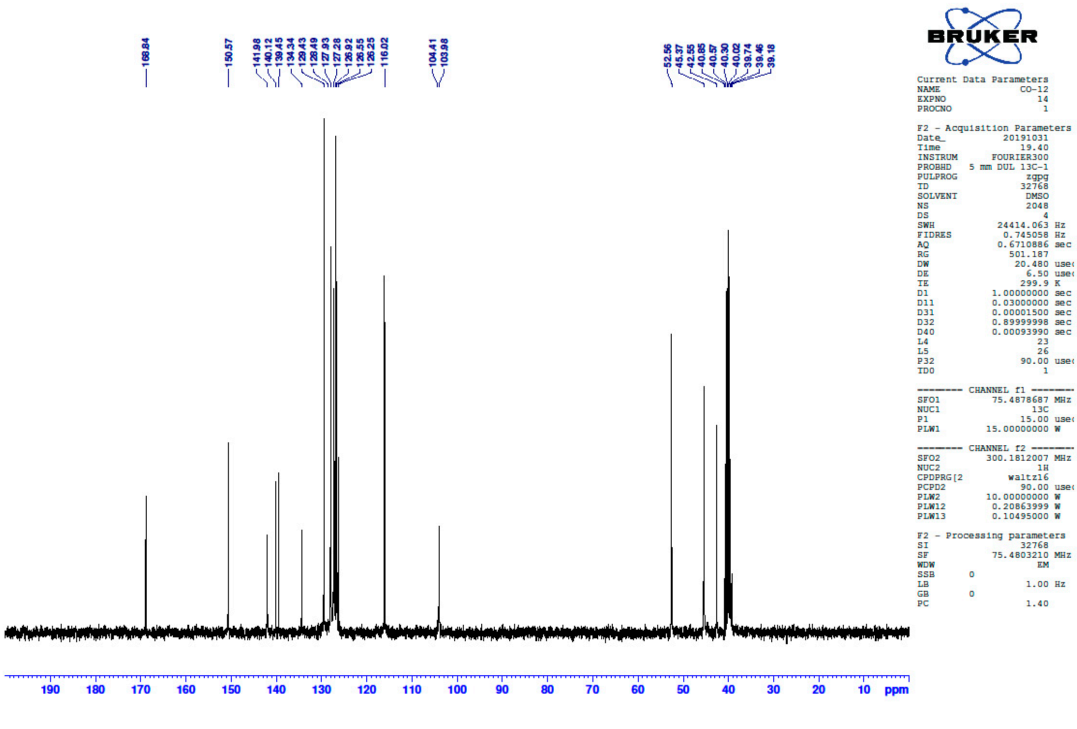

Figure S32. ^{13}C-NMR spectra of compound **3i**

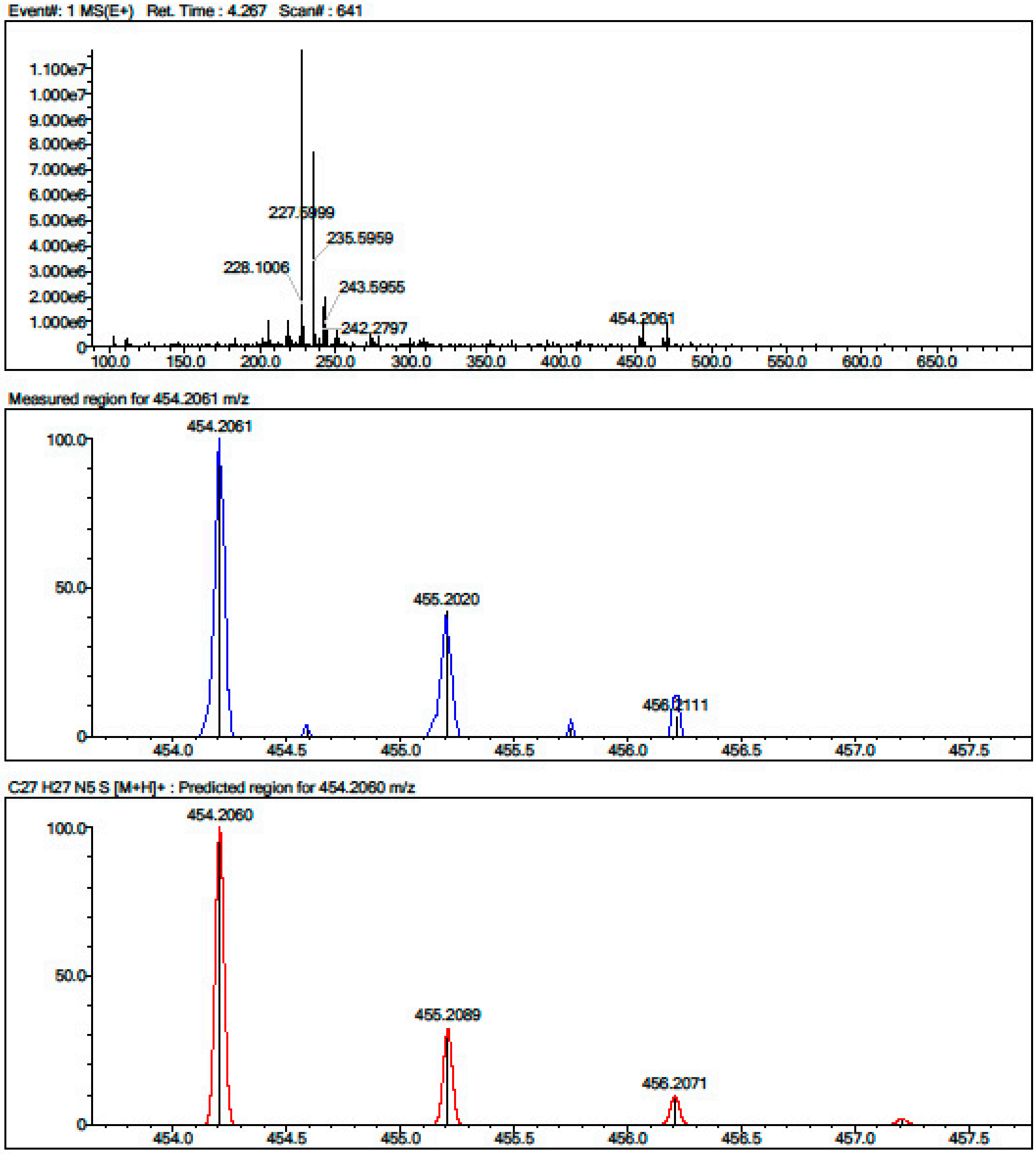

Formula Predictor Report - CO-12_11.lcd

Page 1 of 1

Data File: C:\LabSolutions\Data\Analiz\derya\CO-12_11.lcd

Elmt	Val.	Min	Max	Elmt	Val.	Min	Max	Elmt	Val.	Min	Max	Elmt	Val.	Min	Max	Use Adduct
H	1	0	40	O	2	0	2	S	2	1	1	Ru	2	0	0	H
C	4	21	35	F	1	0	0	Cl	1	0	2	Pd	2	0	0	
N	3	5	6	P	3	0	0	Br	1	0	1	I	3	0	0	

Error Margin (ppm): 20
HC Ratio: unlimited
Max Isotopes: 3
MSn Iso RI (%): 10.00

DBE Range: 10.0 - 20.0
Apply N Rule: yes
Isotope RI (%): 1.00
MSn Logic Mode: AND

Electron Ions: both
Use MSn Info: yes
Isotope Res: 9000
Max Results: 100

Event#: 1 MS(E+) Ret. Time : 4.267 Scan# : 641

Measured region for 454.2061 m/z

C27 H27 N5 S [M+H]+ : Predicted region for 454.2060 m/z

Rank	Score	Formula (M)	Ion	Meas. m/z	Pred. m/z	Df. (mDa)	Df. (ppm)	Iso	DBE
1	67.25	C27 H27 N5 S	[M+H]+	454.2061	454.2060	0.1	0.22	67.25	17.0

Figure S33. HRMS spectra of compound **3i**

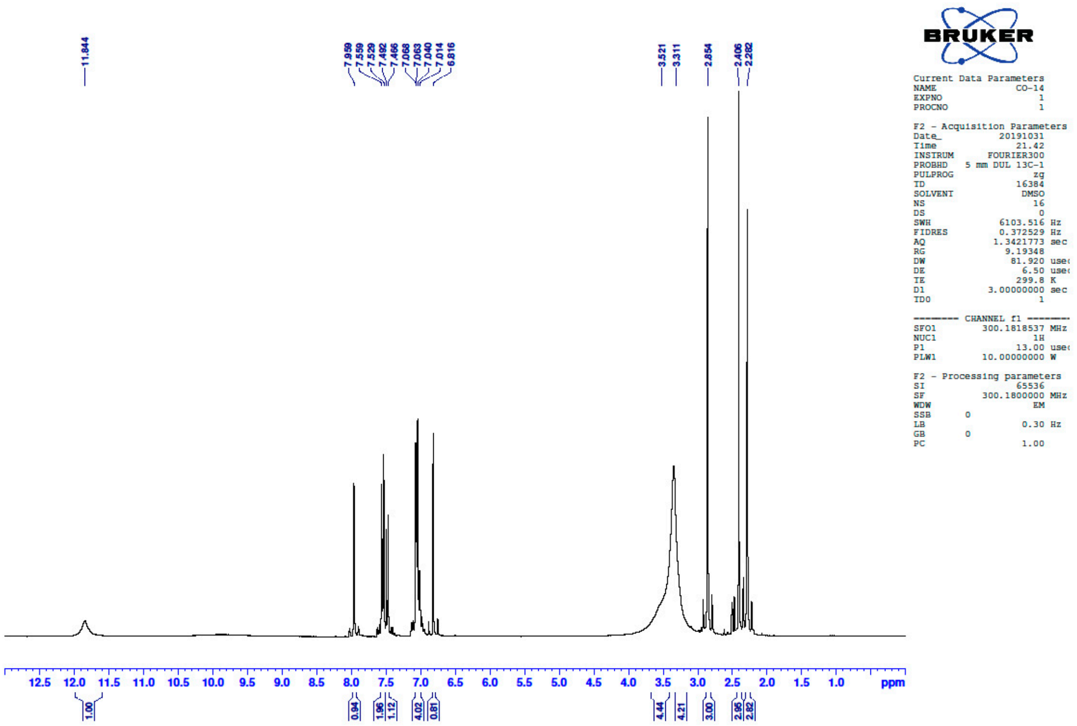

Figure S34. ^{1}H-NMR spectra of compound **3j**

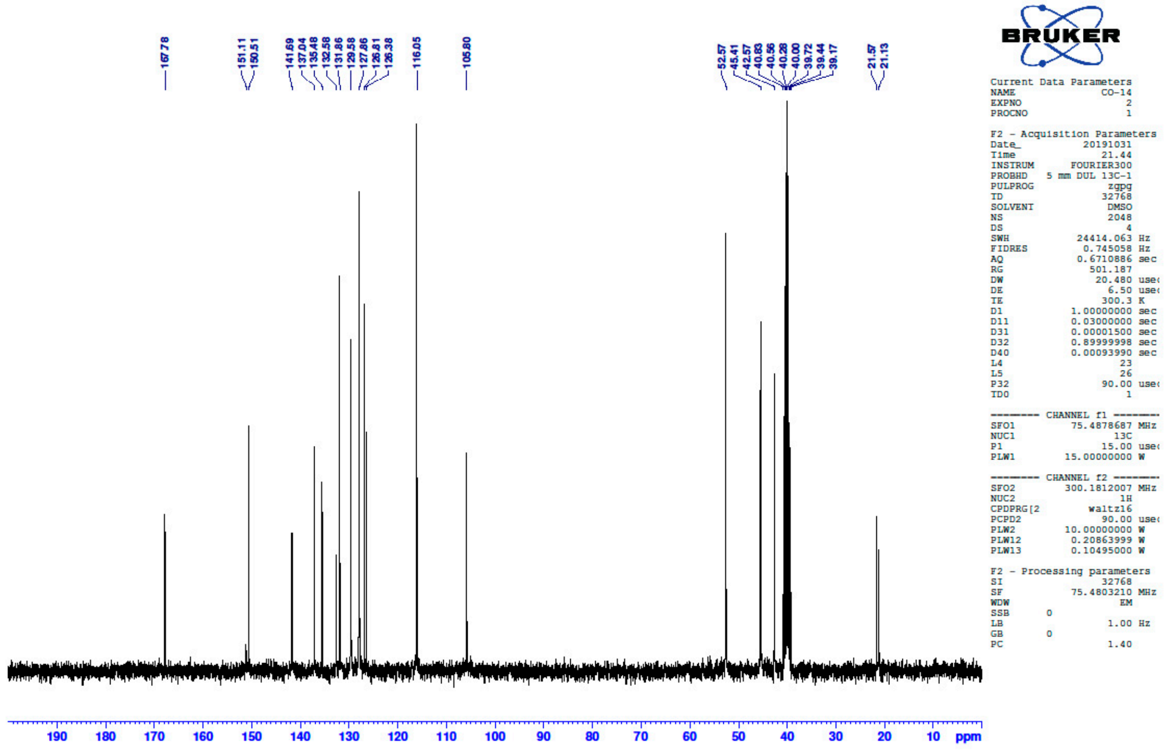

Figure S35. ^{13}C-NMR spectra of compound **3j**

Formula Predictor Report - CO-14_13.lcd Page 1 of 1

Data File: C:\LabSolutions\Data\Analiz\derya\CO-14_13.lcd

Elmt	Val.	Min	Max	Elmt	Val.	Min	Max	Elmt	Val.	Min	Max	Elmt	Val.	Min	Max	Use Adduct
H	1	0	40	O	2	0	2	S	2	1	1	Ru	2	0	0	H
C	4	21	35	F	1	0	0	Cl	1	0	1	Pd	2	0	0	
N	3	5	6	P	3	0	0	Br	1	0	1	I	3	0	0	

Error Margin (ppm):	20	DBE Range:	10.0 - 20.0	Electron Ions:	both
HC Ratio:	unlimited	Apply N Rule:	yes	Use MSn Info:	yes
Max Isotopes:	3	Isotope RI (%):	1.00	Isotope Res:	9000
MSn Iso RI (%):	10.00	MSn Logic Mode:	AND	Max Results:	100

Event#: 1 MS(E+) Ret. Time : 3.427 Scan# : 515

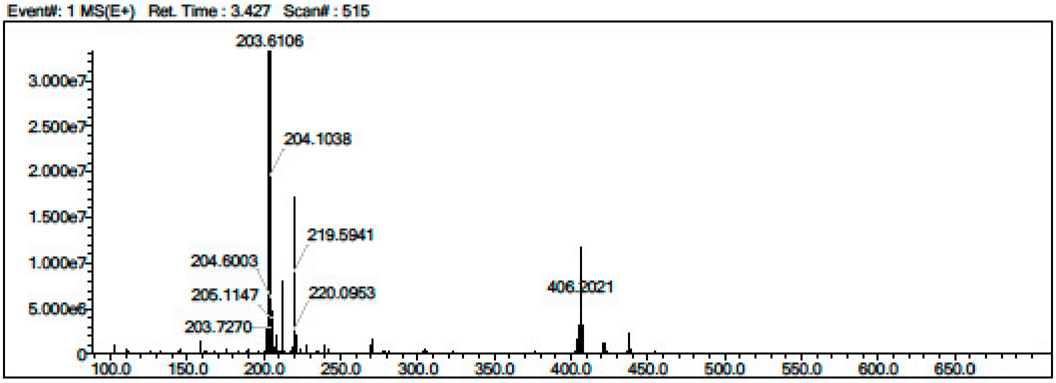

Measured region for 406.2021 m/z

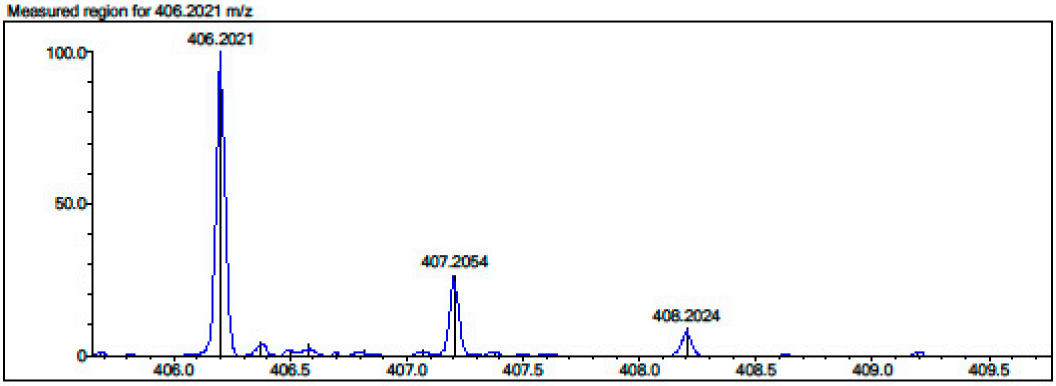

C23 H27 N5 S [M+H]+ : Predicted region for 406.2060 m/z

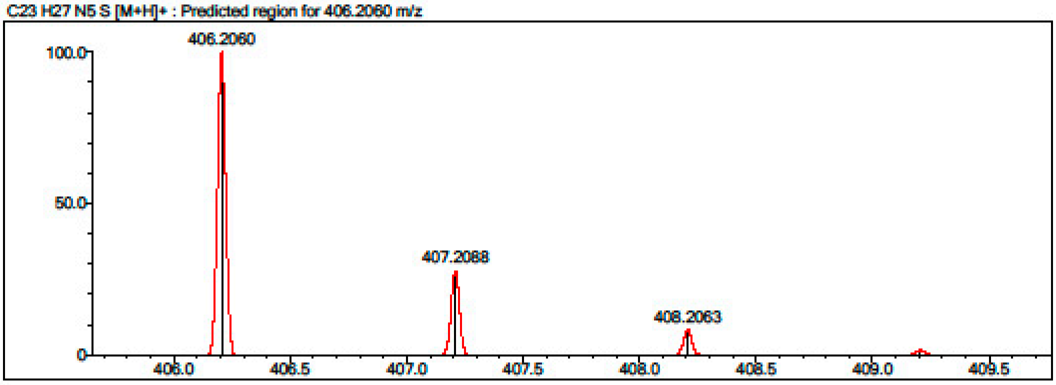

Rank	Score	Formula (M)	Ion	Meas. m/z	Pred. m/z	Df. (mDa)	Df. (ppm)	Iso	DBE
1	23.71	C23 H27 N5 S	[M+H]+	406.2021	406.2060	-3.9	-9.60	53.89	13.0

Figure S36. HRMS spectra of compound **3j**

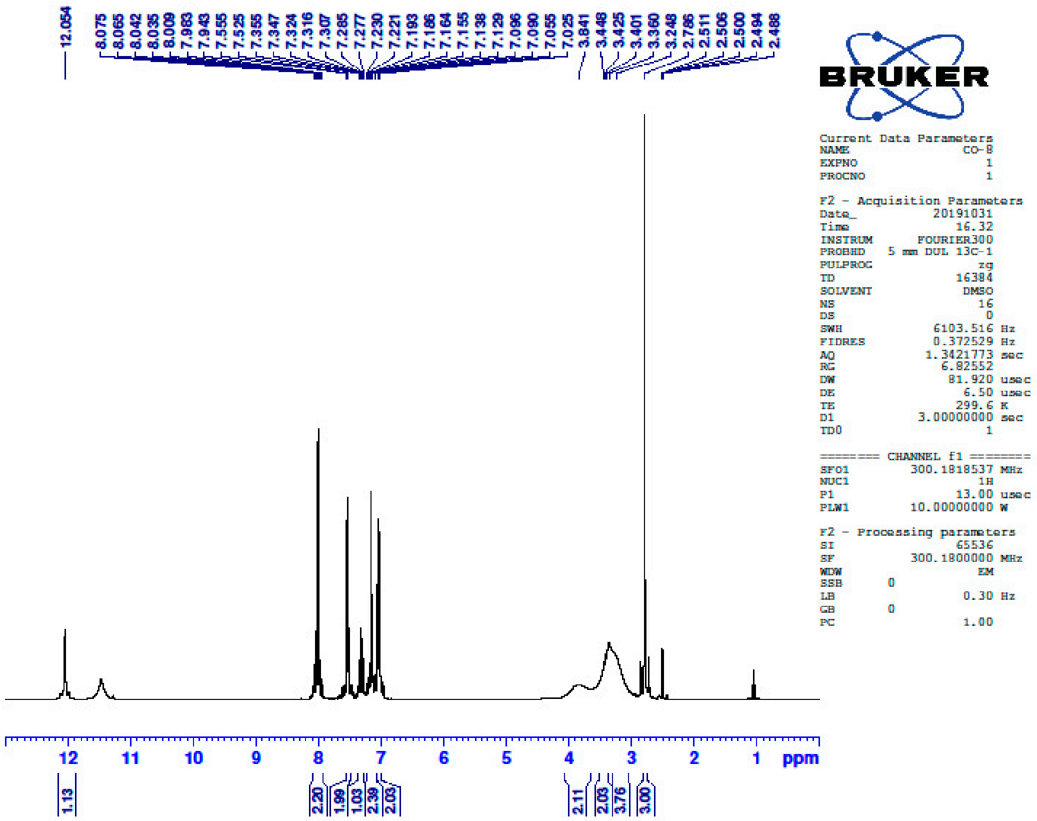

Figure S37. ^{1}H-NMR spectra of compound **3k**

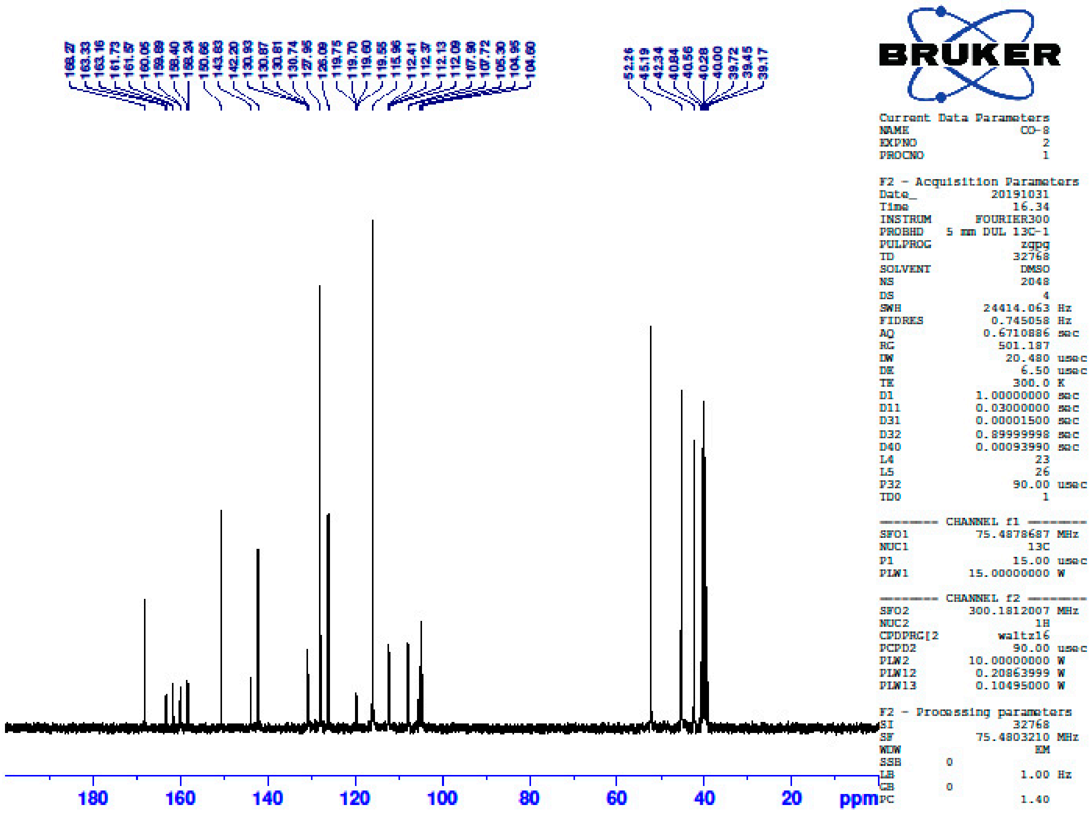

Figure S38. ^{13}C-NMR spectra of compound **3k**

Formula Predictor Report - CO-8_8.lcd Page 1 of 1

Data File: C:\LabSolutions\Data\Analiz\derya\CO-8_8.lcd

Elmt	Val.	Min	Max	Elmt	Val.	Min	Max	Elmt	Val.	Min	Max	Elmt	Val.	Min	Max	Use Adduct
H	1	0	40	O	2	0	2	S	2	1	1	Ru	2	0	0	H
C	4	21	35	F	1	1	2	Cl	1	0	2	Pd	2	0	0	
N	3	5	6	P	3	0	0	Br	1	0	1	I	3	0	0	

Error Margin (ppm): 25
HC Ratio: unlimited
Max Isotopes: 3
MSn Iso RI (%): 10.00

DBE Range: 10.0 - 20.0
Apply N Rule: yes
Isotope RI (%): 1.00
MSn Logic Mode: AND

Electron Ions: both
Use MSn Info: yes
Isotope Res: 9000
Max Results: 100

Event#: 1 MS(E+) Ret. Time : 3.147 Scan# : 473

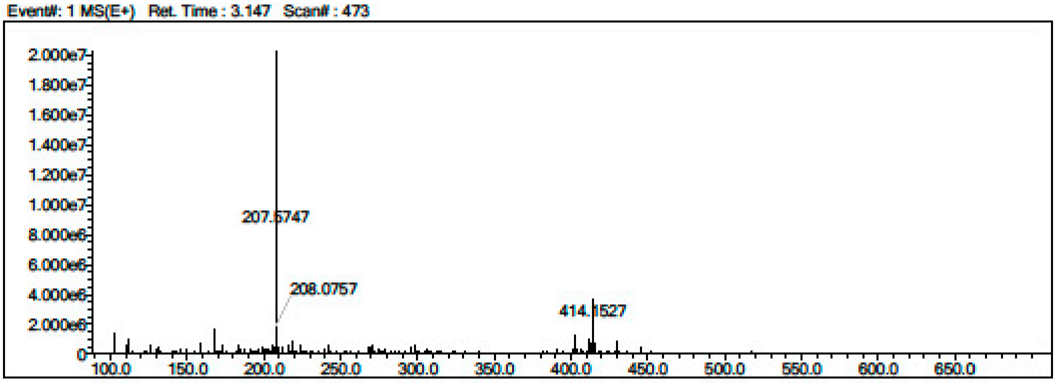

Measured region for 414.1527 m/z

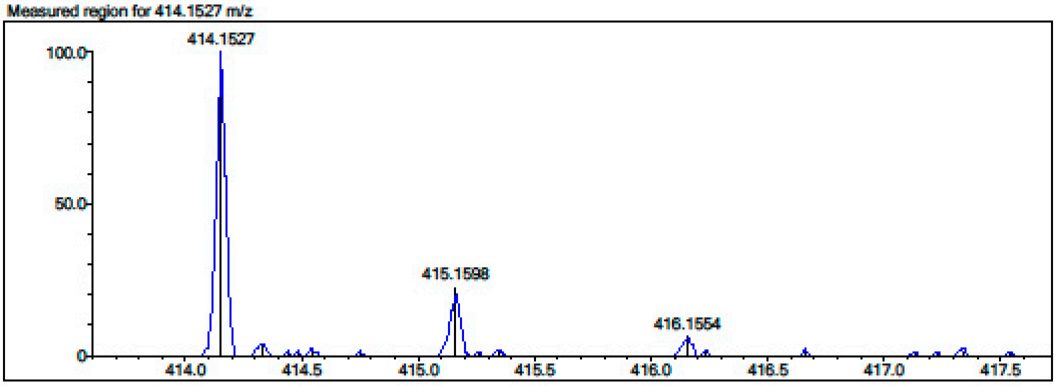

C21 H21 N5 F2 S [M+H]+ : Predicted region for 414.1558 m/z

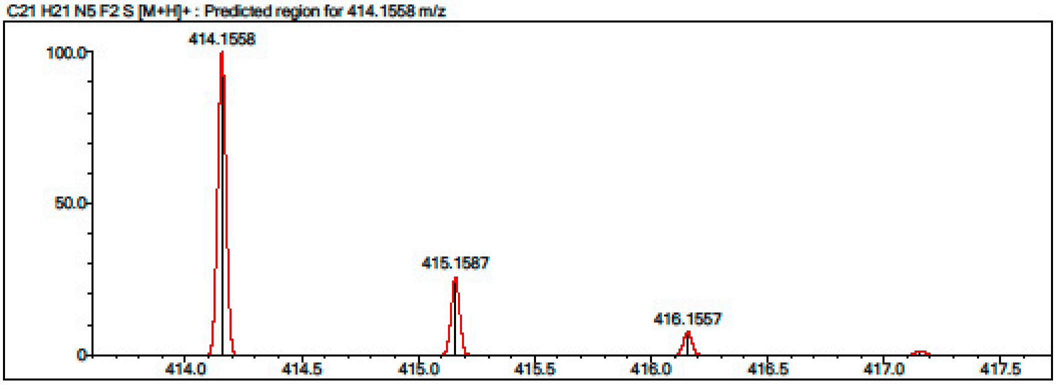

Rank	Score	Formula (M)	Ion	Meas. m/z	Pred. m/z	Df. (mDa)	Df. (ppm)	Iso	DBE
1	42.54	C21 H21 N5 F2 S	[M+H]+	414.1527	414.1558	-3.1	-7.49	65.35	13.0

Figure S39. HRMS spectra of compound **3k**

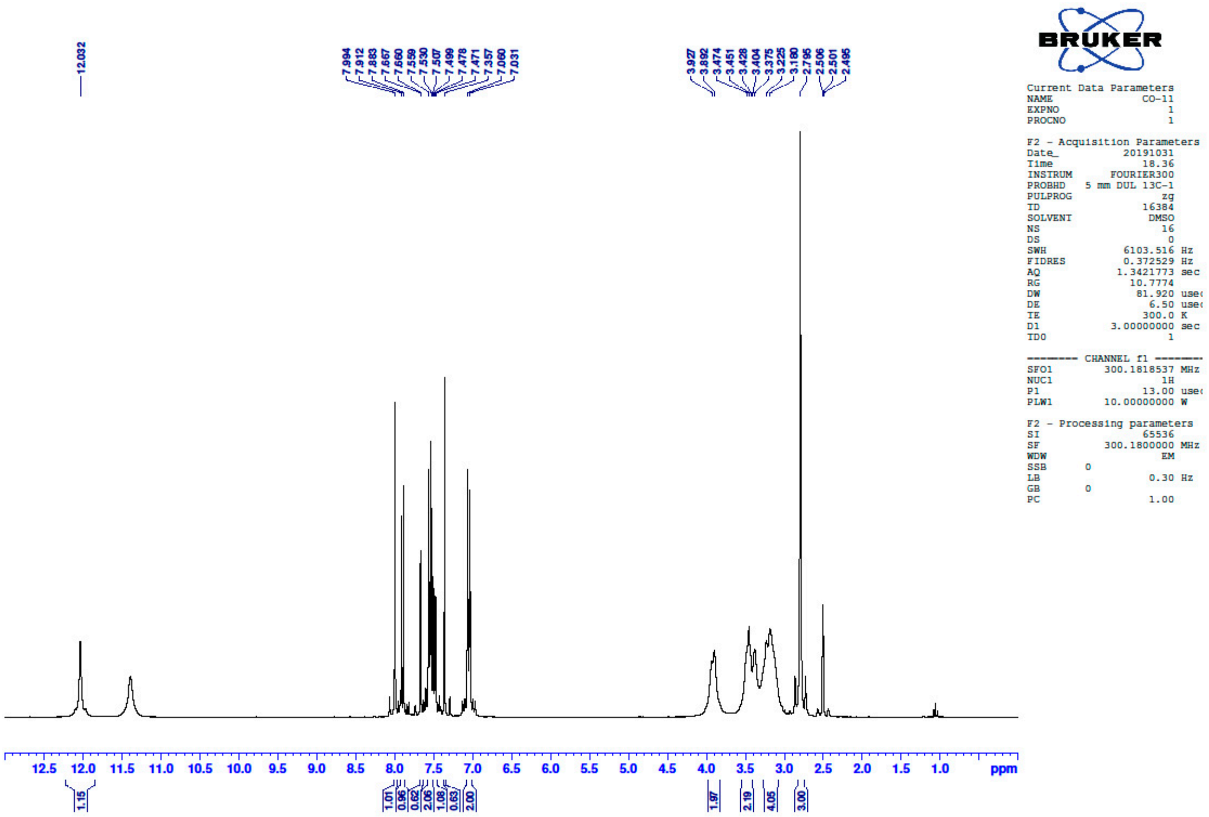

Figure S40. ^{1}H-NMR spectra of compound **3l**

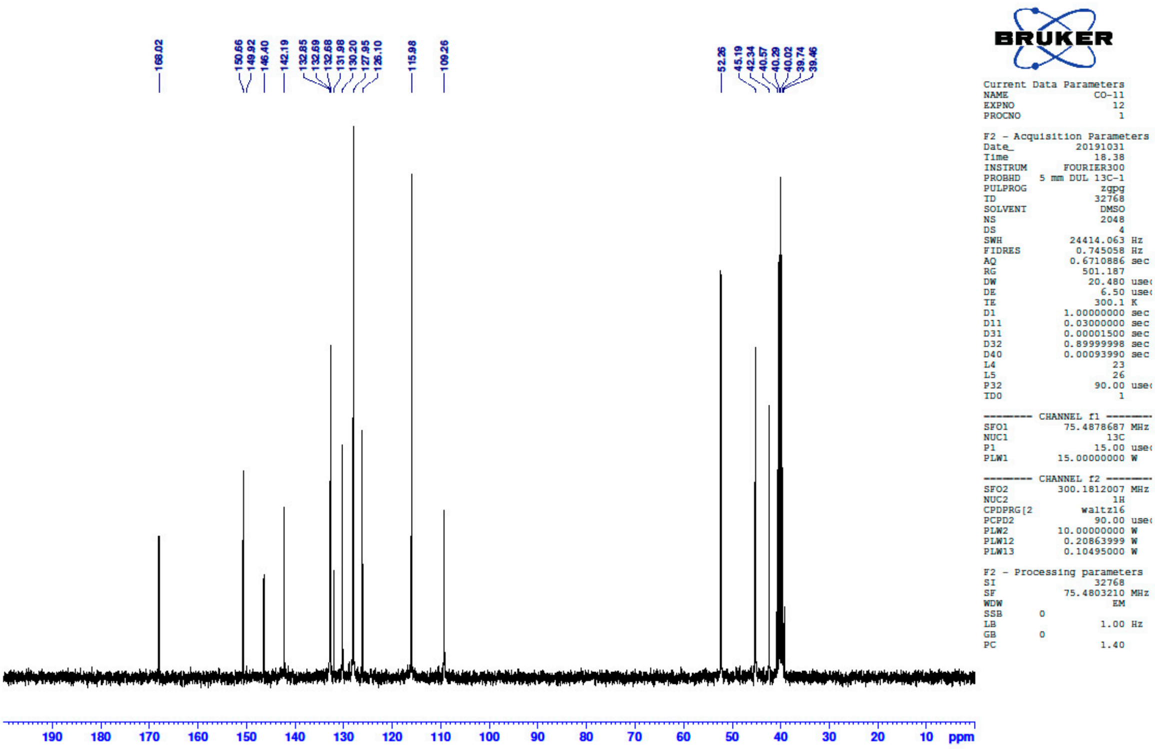

Figure S41. ^{13}C-NMR spectra of compound **3l**

Formula Predictor Report - CO-11_10.lcd

Data File: C:\LabSolutions\Data\Analiz\derya\CO-11_10.lcd

Elmt	Val.	Min	Max	Elmt	Val.	Min	Max	Elmt	Val.	Min	Max	Elmt	Val.	Min	Max	Use Adduct
H	1	0	40	O	2	0	2	S	2	1	1	Ru	2	0	0	H
C	4	21	35	F	1	0	0	Cl	1	0	2	Pd	2	0	0	
N	3	5	6	P	3	0	0	Br	1	0	1	I	3	0	0	

Error Margin (ppm): 20
HC Ratio: unlimited
Max Isotopes: 3
MSn Iso RI (%): 10.00

DBE Range: 10.0 - 20.0
Apply N Rule: yes
Isotope RI (%): 1.00
MSn Logic Mode: AND

Electron Ions: both
Use MSn Info: yes
Isotope Res: 9000
Max Results: 100

Event#: 1 MS(E+) Ret. Time : 3.347 Scan# : 503

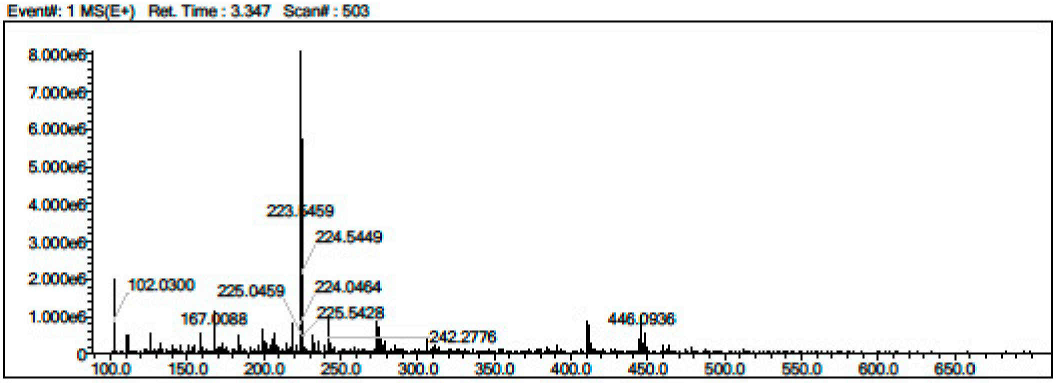

Measured region for 446.0936 m/z

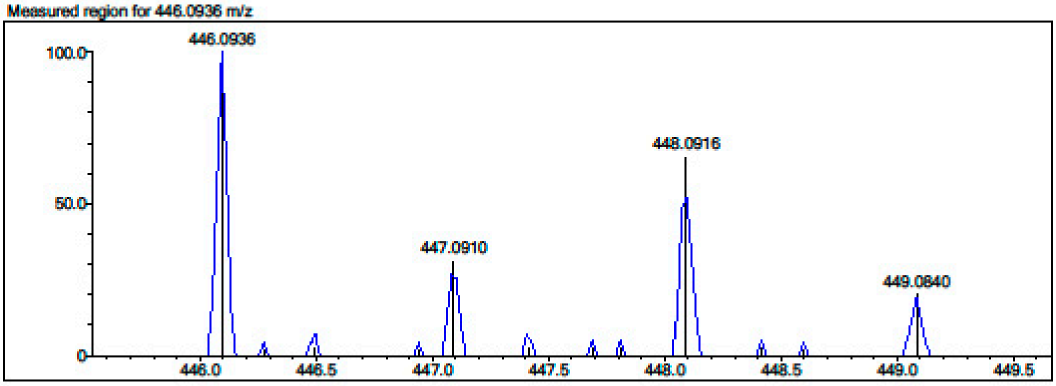

C21 H21 N5 S Cl2 [M+H]+ : Predicted region for 446.0967 m/z

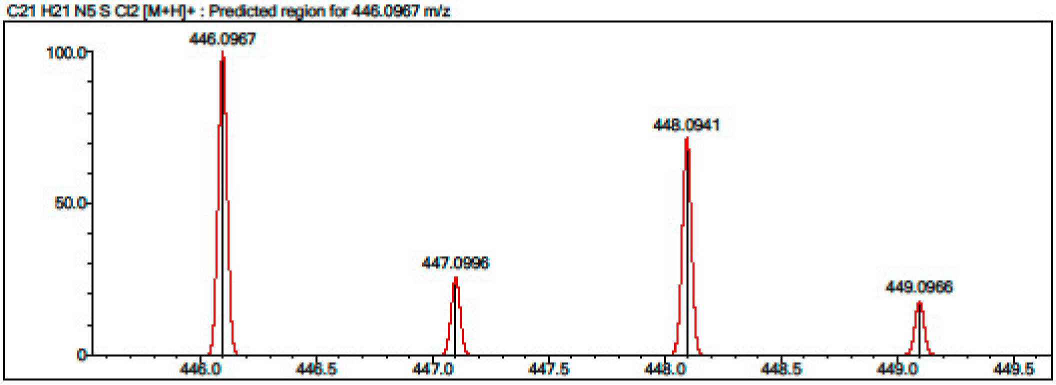

Rank	Score	Formula (M)	Ion	Meas. m/z	Pred. m/z	Df. (mDa)	Df. (ppm)	Iso	DBE
1	29.48	C21 H21 N5 S Cl2	[M+H]+	446.0936	446.0967	-3.1	-6.95	41.81	13.0

Figure S42. HRMS spectra of compound **3l**
